# Filamentation Involves Two Overlapping, but Distinct, Programs of Filamentation in the Pathogenic Fungus *Candida albicans*

**DOI:** 10.1534/g3.117.300224

**Published:** 2017-09-25

**Authors:** Jahaun Azadmanesh, Austin M. Gowen, Paul E. Creger, Nichole D. Schafer, Jill R. Blankenship

**Affiliations:** * Biology Department, University of Nebraska Omaha, Nebraska 68182; † Department of Biochemistry and Molecular Biology, University of Nebraska Medical Center, Omaha, Nebraska 68198

**Keywords:** *Candida albicans*, filamentation, hyphal growth, *in vitro* model comparisons

## Abstract

The ability of the human pathogenic fungus *Candida albicans* to switch between yeast-like and filamentous forms of growth has long been linked to pathogenesis. Numerous environmental conditions, including growth at high temperatures, nutrient limitation, and exposure to serum, can trigger this morphological switch and are frequently used in *in vitro* models to identify genes with roles in filamentation. Previous work has suggested that differences exist between the various *in vitro* models both in the genetic requirements for filamentation and transcriptional responses to distinct filamentation-inducing media, but these differences had not been analyzed in detail. We compared 10 *in vitro* models for filamentation and found broad genetic and transcriptomic differences between model systems. The comparative analysis enabled the discovery of novel media-independent genetic requirements for filamentation as well as a core filamentation transcriptional profile. Our data also suggest that the physical environment drives distinct programs of filamentation in *C. albicans*, which has significant implications for filamentation *in vivo*.

*Candida albicans* is a major pathogen of humans, causing ∼20,000 invasive infections per year in the United Stated alone (CDC, 2013). Mortality rates for *Candida* infections can be as high as 40%, and these rates have remained static as mortality rates for less prevalent fungal infections have declined (Pfaller and Diekema 2007). *C. albicans* is also a constituent of the human microbiome and can be found in the gastrointestinal and genitourinary tracts of 30–70% of healthy adults (Kleinegger *et al.* 1996). Invasive infections generally arise from overgrowth of *C. albicans* within a susceptible patient’s own microbiome, but the conversion from commensal to pathogen is not well understood. It is clear that morphological transitions of the fungus as well as host immune factors play a part in this conversion.

One of the defining characteristics of *C. albicans* infection is its ability to transition between several morphological forms, and these forms impart distinct properties important for *in vivo* survival and pathogenesis. While *C. albicans* can exist in numerous cellular forms [reviewed in Noble *et al.* (2017)], the most relevant forms for infection appear to be elongated filamentous cells and rounded yeast-like cells. Yeast-like and filamentous cells not only vary in their physiology, but they also have distinct cell wall compositions and cell surface proteins, which have key roles in pathogenesis and immune cell recognition. Differing glucan compositions of yeast-like and filamentous cells drive diverse immune responses to each cell type (Lowman *et al.* 2014). For instance, macrophage recognition and activation of the TH17 response to invading *C. albicans* appear to depend on filament-specific glucans (Cheng *et al.* 2011; Lowman *et al.* 2014). Differing surface and secreted molecules also change how *C. albicans* interacts with its human host. The filament-specific adhesins Hwp1 and Als3 increase adhesion to host cells and induce endocytosis of *C. albicans*, while the yeast-specific protein Ywp1 appears to have an antiadhesive effect and promotes dispersal of yeast form cells (Staab *et al.* 1999; Granger *et al.* 2005; Phan *et al.* 2007). In addition, the filament-specific toxin, Candidalysin, permeabilizes epithelial cell membranes and is vital for mucosal infection (Moyes *et al.* 2016). The distinct properties of each cell type must contribute to pathogenesis, as cells that are unable to undergo the switch between yeast and filamentous growth *in vitro* are unable to establish a systemic infection (Lo *et al.* 1997). In addition, cells locked in either a yeast-like or filamentous state following infection are unable to cause significant disease in animal models (Murad *et al.* 2001). Thus, both morphologies are vital for pathogenesis in this organism.

Regulation of filamentation is a complex process that can be triggered by a variety of environmental conditions. Much of the signaling for filamentation funnels through the cAMP and Ste20 MAPK pathways downstream of a variety of environmental cues, including temperature, nitrogen starvation, serum, the quorum sensing factor farnesol, and CO_2_ (Sudbery 2011). Alkaline environments, on the other hand, appear to trigger filamentation via the Rim101 pathway (Davis *et al.* 2000; Martin *et al.* 2010). These pathways activate transcriptional activators that regulate genes involved in the initiation and maintenance of filamentation. *C. albicans* filamentation models suggest that the diverse signaling events triggering filamentation all converge on a single response to build and maintain the filamentous state.

Our understanding of the genetic requirements for filamentation is based on work using a variety of *in vitro* models that are meant to mimic the varied environmental filamentation triggers. However, variability has been noted between these model systems in both the requirements for filamentation and in the transcriptional response to filamentation. Most of the variable observations have been made in single gene assays, but some reports have shown hints of larger variation in genetic requirements for filamentation and very distinct transcriptional responses to filamentation in differing inducing conditions (Martin *et al.* 2013; O’Meara *et al.* 2015). As a field, however, we have not taken a systematic approach to compare *in vitro* filamentation conditions and have little data on how divergent *C. albicans* responses may be in these distinct environments. Thus, the goal of our study was to compare filamentation in diverse *in vitro* models to identify patterns underlying filamentation across conditions that elucidate conserved features of *C. albicans* biology.

We took a dual approach to characterize filamentation in *C. albicans*. To gain a sense of genetic requirements across varied filamentation conditions, we screened 124 mutant *C. albicans* strains for their ability to filament in 10 distinct *in vitro* conditions. To discern patterns underlying genetic responses to filamentation, we examined the transcriptional response of a wild-type strain to the same 10 *in vitro* conditions. The results of our study have led us to three main conclusions. First, there is wide variability in the genes required for filamentation in inducing conditions, and few mutant strains exhibit filamentation defects across the majority of conditions tested. Second, gene expression also varies widely across conditions, and only a small subset of genes are differentially regulated across all conditions. Finally, both the genetic screen and the transcriptional analysis suggest that filamentation in solid and liquid media represent distinct programs of filamentation, which is a fundamental shift in our understanding of the process and has implications for both pathogenesis and treatment.

## Materials and Methods

### Strains and media

Strains were grown in yeast extract-peptone-dextrose (YPD) noninducing media, prepared as previously described (Sherman 1991), 10% fetal bovine serum (FBS) media (10% FBS with 2% dextrose), 10% FBS media with YPD (10% FBS, 10 g yeast extract, 20 g peptone, 2% dextrose, and H_2_O for a final volume of 1 L), Lee’s media (Lee *et al.* 1975), RPMI media with 2.1 mM L-glutamine and buffered with 165 mM MOPs, and Spider medium (10 g D-mannitol, 10 g nutrient broth, 2 g K_2_HPO_4_, in 1 L of H_2_O). For solid media, 16 g of agar (RPI) was added per liter of media.

Wild-type strain SC5314 and marker-matched strain SN250 (Noble and Johnson 2005) were used as controls throughout. Mutant strains (Supplemental Material, Table S6) were selected from the Noble deletion collection (Noble *et al.* 2010) obtained from the Fungal Genetics Stock Center (Manhattan, KS).

### Filamentation analysis

Cells were grown overnight in 3 ml YPD media at 30° with shaking. One-milliliter aliquots of overnight cultures were centrifuged at top speed in a microcentrifuge and washed twice with an equal volume of phosphate buffered saline (PBS) at pH 7.2. Washed cells were resuspended in an equal volume of PBS. For solid filamentation phenotypic assays, 1 µl of resuspended cells were plated in a grid on noninducing (YPD) and inducing (FBS, Lee’s, RPMI, Spider) agar plates. Noninducing plates were incubated at 30° and inducing plates were incubated at 37°. All strains were tested in triplicate and the triplicates were plated on different agar plates. Colonies and colony edges were imaged 4–5 d postincubation. Colony edges were imaged on a Evos FL inverted microscope at 4× magnification. For liquid filamentation assays, 10 µl of washed cells was added to 2 ml of prewarmed media in a glass-bottom microscopy dish (MatTak). Incubated cells were grown in the microscopy dishes, with shaking, at 37° for 3 hr and imaged on a Zeiss Axiovision microscope at 20× magnification. For liquid YPD assays, 10 µl of the washed overnight cells were imaged on a glass slide. Five images were taken for all inducing conditions and 3–5 images were taken for the noninducing condition (File S1).

**Figure 1:**
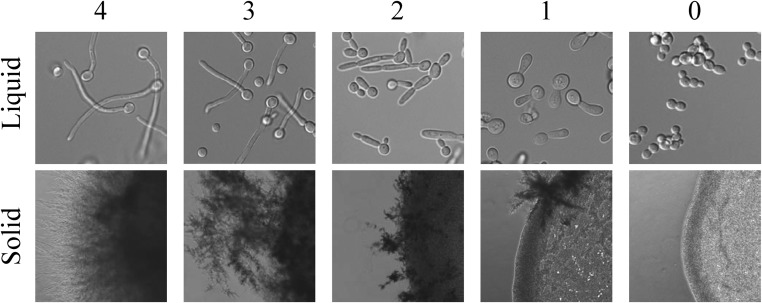
Filamentation scoring range for liquid and solid filamentation conditions. *C. albicans* wild-type and 124 mutant strains were tested for their ability to filament in liquid and solid filamentation conditions. For both assays, cells grown overnight in YPD were centrifuged and washed 2× with PBS prior to the experiment. For the liquid assays, 10 µl of washed cells were added to a prewarmed glass-bottom microscopy dish with inducing media. Cells were imaged after 3 hr of incubation at 37° with shaking. For solid assays, 1 µl of washed cells was plated onto solid media and grown at 37° for 4–5 d before imaging. The images shown are representative images from the analysis at the respective scores for inducing conditions. The scoring range for Spider solid media is shown in Figure S7.

Phenotypes of mutant strains were compared to the control strains SC5314, a wild-type strain, and SN250, a marker-matched control strain for the deletion strain set. Strains were scored from 0 (completely abnormal) to 4 (similar to controls). Thus, in filament-inducing conditions, a score of four represented a strain with no observable filamentation defect, while in noninducing conditions, a score of four represented a strain growing in a yeast-like state (Figure 1 and Figure S1, Figure S2, Figure S3, Figure S4, Figure S5, Figure S6, Figure S7, Figure S8, Figure S9, and Figure S10). Liquid assays were scored from microscopic images of cells in culture, while solid assays were scored by observing filaments extending from the edge of the fungal colony with the exception of Spider plates (Figure S7). Spider plates were scored by the colony phenotype on the plate due to significant issues in the reliability of filaments extending from the colony in control cells. For the purposes of our analysis, we considered scores of 2.5 or below to represent a significant filamentation defect. Phenotypes were scored by three independent scorers and the final scores were averaged.

### RNA extraction and cDNA generation

SC5314 cells were grown overnight in YPD media at 30° with shaking. Cells were washed twice and resuspended with equal volumes of PBS. For liquid conditions, 100 µl of washed cells was incubated in 50 ml of prewarmed media in a 250-ml flask and grown for 3 hr at 37° (inducing) or 30° (noninducing). Cells were harvested by filtration as previously described (Blankenship *et al.* 2010). For solid conditions, 140 µl of cells and 200 µl of H_2_O were spread on the surface of prewarmed agar plates using 3.5-mm glass beads. Plates were incubated at 37° (inducing) or 30° (noninducing) for 3 hr, and cells were harvested as described in Creger and Blankenship (2017). Harvested cells were centrifuged in a microcentrifuge and frozen at −80°.

RNA was extracted from frozen cells using an RNeasy kit with on-column DNase treatment (Qiagen). RNA quality (260/280 ratios) was measured on a Nanodrop machine.

### RNAseq analysis

RNASeq libraries were generated beginning with 1.8 ng of total RNA following standardized protocols with the TruSeq RNA v2 kit (Illumina, San Diego, CA). Libraries were diluted to a concentration of 6.0 pmol and sequenced on a HiSeq2500 (Illumina) and 100 bp single reads were generated. Total reads and percentages of mapped reads are detailed in Table S1.

### Bioinformatics analysis

All eight *Candida* chromosomes were downloaded from NCBI, and annotation was downloaded into a gff3 file and transformed into a gtf file using gffread. Fastq files were generated using the bcl2fastq software, version 1.8.4. The fastq files for each sample were analyzed using the Tuxedo pipeline in order to find differentially expressed genes. Read alignment was performed using tophat version 2.0. FPKM values were calculated with cufflinks 2.2. The cuffmerge and cuffdiff software were used to calculate fold change values between sets of samples. A P-value of 0.05 was used to differentiate between statistically significant and insignificant genes. FPKM (Fragments Per Kilobase per Million mapped reads) values of each set were normalized to FPKM values from the liquid YPD media samples.

### Clustering analyses and statistics

Filamentation and gene expression data were clustered using the Multiple Experiment Viewer (mev.tm4.org) using hierarchical clustering with a Pearson correlation metric with complete linkage clustering. Approximately unbiased (AU) P values were calculated for both the filamentation and gene expression analysis using the pvclust package (Suzuki and Shimodaira 2006) on R Studio, Inc. (2016) using complete hierarchical clustering and the correlation-based dissimilarity matrix with 10,000 bootstrap replications.

### Data availability

The raw data discussed in this paper have been deposited in NCBI’s Gene Expression Omnibus (Edgar *et al.* 2002) and are accessible through GEO Series accession number GSE99902 (https://www.ncbi.nlm.nih.gov/geo/query/acc.cgi?acc=GSE99902).

## Results

### A comparative phenotypic screen for filamentation demonstrates widely divergent filamentation phenotypes

Phenotypic differences in *C. albicans* filamentation have been noted anecdotally between mutant strains tested in distinct inducing conditions, which suggests that some of the genetic requirements for the initiation and maintenance of filamentation vary between conditions. For instance, the protein kinase A ortholog Tpk1 is required for filamentation on solid media, while the other protein kinase A ortholog TPK2 is required for filamentation in liquid media (Bockmuhl *et al.* 2001). The transcription factor Efg1, whose activity is dependent on protein kinase A activation, is required for filamentation in most conditions tested, but is not required for filamentation in an oral model of infection or during filamentation in embedded conditions (Riggle *et al.* 1999). In addition, many of the strains tested in this work had differing filamentation phenotypes on solid Spider media at 30° *vs.* liquid media containing FBS at 37° (Noble *et al.* 2010). However, it is unclear if these differences are restricted to a few condition-specific genes or if larger differences exist between induction conditions.

To compare genetic requirements for filamentation, we analyzed the filamentation of 124 *C. albicans* deletion strains previously shown to have filamentation defects in solid Spider media at a low induction temperature (Noble *et al.* 2010) in 10 distinct media conditions. Filamentation was examined in solid and liquid versions of 10% FBS, Lee’s media, RPMI media, and Spider media, with noninducing media (YPD) at a noninducing temperature as a control (Figure 1 and Figure S1, Figure S2, Figure S3, Figure S4, Figure S5, Figure S6, Figure S7, Figure S8, Figure S9, and Figure S10).

**Figure 2:**
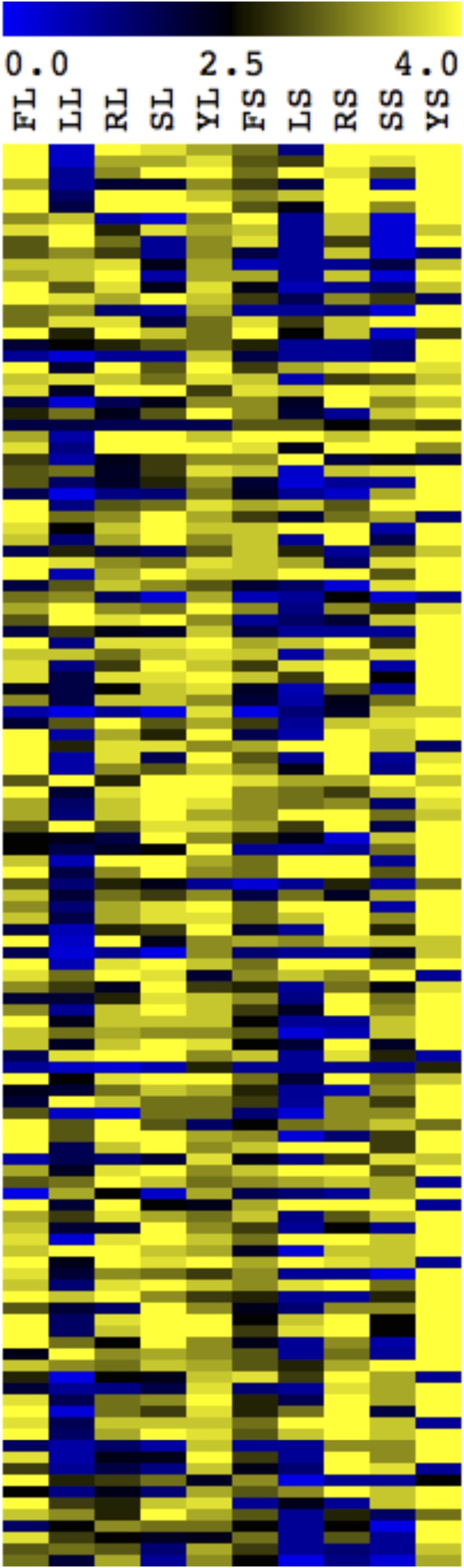
Mutant cells display variation in their ability to filament in distinct inducing conditions. Mutant strains were scored for their phenotype in filament-inducing and noninducing media. Strains were scored from 4 (bright yellow) representing the wild-type phenotype, to 0 (bright blue) representing a mutant phenotype in each respective media. Filament-inducing conditions included liquid or solid FBS (FL and FS, respectively), liquid or solid Lee’s (LL and LS, respectively), liquid or solid RPMI (RL or RS, respectively), and liquid or solid Spider (SL and SS, respectively). Scores of 4 (bright yellow) in these conditions represented wild-type filamentation and scores of 0 (bright blue) represented afilamentous cells. Noninducing conditions, YL and YS (liquid and solid YPD), were scored from 4 (bright yellow) representing a wild-type, nonfilamentous phenotype to 0 (bright blue) representing fully filamentous cells. The score heatmap has distinct conditions shown in columns and individual mutant strain scores in rows. Score details can be found in Table S2.

While the majority of the tested strains (122 of 124 deletion strains) had a filamentation defect in a least one condition in our assay, we observed a great deal of phenotypic variability between mutant strains and also within individual strains tested in differing inducing conditions (Figure 2 and Table S2). This suggests that distinct inducing conditions have very different genetic requirements to initiate and/or maintain filamentation. Indeed, 32 mutant strains exhibited a strong filamentation defect (a score of 2.5 or less) in only one condition (Table S2). The majority of singleton phenotypic defects were observed in either solid or liquid Lee’s media, which had the highest numbers of strains with phenotypic defects overall. Generally, the filamentation results across media types suggest that genetic requirements for the initiation or maintenance of filamentation are distinct between inducing conditions.

### Strains with broad defects may have media-independent roles in filamentation

While many strains did not exhibit consistent defects in filamentation across the panel of conditions tested, we noted that 17 strains had severe defects across most, if not all, of the inducing conditions (Table 1). Two of the strains in this group of highly defective strains, containing mutations in *rim101Δ/Δ* and *gpa2Δ/Δ*, have well-documented roles in pathways important for triggering pathogenesis. Rim101 responds to extracellular pH and triggers expression of filamentation-specific genes by activation of the transcription factor Efg1 (Davis *et al.* 2000; Li *et al.* 2004). Gpa2 is a G protein α-subunit that is part of the cAMP signaling pathway, one of the main environmental response pathways for filamentation (Maidan *et al.* 2005). The broad defect that strains bearing deletions in these genes had across distinct filamentation conditions was anticipated, and suggests that other genes with broad defects across conditions also have vital roles in filamentation.

**Table 1:** Mutant strains with broad, severe filamentation defects

		Liquid*^a^*				Solid*^a^*			
ORF*^b^*	Gene*^b^*	F	L	R	S	F	L	R	S
C1_08990C	KEX2	0.00	0.50	0.33	0.67	0.00	0.00	0.00	1.00
C3_02960C	KRE5	0.00	0.33	0.00	0.00	1.83	0.00	0.00	1.33
C4_00610W		1.67	0.33	1.00	0.17	1.33	1.00	0.00	2.17
CR_03430W		0.83	0.17	0.00	0.17	0.17	1.33	2.17	3.67
C6_02740W		3.17	3.33	1.33	0.00	0.00	0.00	1.33	0.33
C1_04630C		1.67	0.17	1.17	1.33	2.17	0.00	0.50	3.50
C3_02240C	GPA2	1.83	2.83	2.33	2.17	1.83	0.00	0.00	0.00
C1_14340C	RIM101	1.50	0.83	1.50	0.83	0.00	0.00	3.33	3.33
C2_01620W	COX4	3.17	3.33	1.50	0.33	0.67	0.00	2.50	0.33
CR_07580C	TSC11	3.00	1.50	2.17	2.67	2.00	0.33	0.00	1.00
C6_03920W	SNF4	3.17	2.00	3.50	0.00	2.50	0.17	1.17	0.33
C4_04530C	PHR1	2.50	1.83	2.17	2.50	0.00	0.83	0.00	3.17
C4_04090C		0.17	3.50	2.50	0.50	1.67	1.33	0.00	3.50
C3_03880C	PEP8	2.00	0.00	1.17	1.83	1.33	1.00	3.83	3.50
CR_02640W	RFG1	1.50	2.50	3.33	3.17	2.50	0.00	2.50	0.17
C1_07970C	IRE1	0.83	1.67	1.33	2.00	2.33	1.17	2.83	3.83
CR_07090W	STT4	2.33	1.83	2.50	3.67	2.17	0.67	3.00	0.83

a Average filamentation scores from three independent scorers in the indicated strains in liquid and solid conditions. F (FBS), L (Lee’s media), R (RPMI-MOPs media), or S (Spider media). Darkened boxes indicate filamentation scores 2.50 and below.

b ORF (open reading frame) and Gene refer to the genes mutated in the indicated strain.

The broad defects of the 15 additional strains with significant filamentation defects in at least six of eight tested conditions suggest that these genes also have vital, general roles in the initiation or maintenance of filamentation. Only three of these 15 strains, containing mutations in *kre5Δ/Δ*, *kex2Δ/Δ*, and *C4_00610WΔ/Δ*, had filamentation defects across all media (Table 1). Mutations in *cox4Δ/Δ*, *ire1Δ/Δ*, *pep8Δ/Δ*, *phr1Δ/Δ*, *rfg1Δ/Δ*, *snf4Δ/Δ*, *stt4Δ/Δ*, *tsc11Δ/Δ*, *CR_03430WΔ/Δ*, *C1_04630CΔ/Δ*, *C4_04090CΔ/Δ*, and *C6_02740WΔ/Δ* exhibited significant defects in filamentation in at least six conditions. While mutations in these genes were previously known to affect filamentation, their precise role in the process has not been described. Additional work will be needed to either tie these genes to existing filamentation pathways or identify novel pathways in which these genes participate.

### Correlation of filamentation to virulence

The ultimate goal of *in vitro* filamentation assays is to predict filamentation, and thus pathogenesis, *in vivo*. Given the varied phenotypes observed in the *in vitro* filamentation assays, we wanted to investigate whether filamentation defects in any of the *in vitro* conditions were more highly associated with virulence defects. All of the strains in the deletion set we analyzed were previously tested for competitive survival *in vivo* in pooled sets, which could identify strains with survival defects in a murine tail vein injection model of infection (Noble *et al.* 2010). A total of 42 strains in our assay demonstrated survival defects in those pooled infection assays (Table S3). We also identified deletion mutations analogous to 27 of the deletion mutations in our set that had been tested for virulence in single-strain assays using the same model of infection (Skrzypek *et al.* 2017). Thirteen of the 27 strains with single-strain virulence defects did not exhibit survival defects in pooled infection assays (Table S3). For the purpose of our analysis, we designated strains with competitive growth defects and/or single-strain virulence defects in analogous mutant strains as virulence defective strains, which accounted for 55 of the 124 strains tested.

We compared average filamentation scores of strains in each inducing condition with known virulence defects, as defined above, with average filamentation scores for strains with no known virulence defects in the same condition (neither a competitive defect or known defect in single-strain virulence assays) to determine whether filamentation in certain conditions might be predictive of virulence defects. Only two conditions, solid Lee’s and solid RPMI, showed a statistically significant reduction in filamentation scores in strains with virulence defects as defined above (Table 2). The strongest defect was in solid RMPI media, where the average score of virulence defective strains was >1 point lower than the strains with no known virulence defects. While this analysis is not completely definitive, our hypothesis is that filamentation defects in solid RPMI medium are predictive of filamentation defects *in vivo*, which is directly contributing to virulence defects in these mutant strains.

**Table 2:** Average filamentation scores and virulence

State*^a^*	Virulence*^b^*	FBS	Lee’s	RPMI	Spider	YPD
Liquid	Defect	3.06	2.66*^c^*	3.12	2.89	3.39
Liquid	WT	3.35	1.74*^c^*	2.96	3.12	3.53
Solid	Defect	2.70	1.49*^c^*	2.25*^c^*	2.62	3.50
Solid	WT	2.81	2.08*^c^*	3.31*^c^*	2.56	3.57

a State refers to liquid or solid assays.

b Virulence refers to murine tail vein injection models of systemic infection. Values in each condition in the “defect” category represent the average filamentation score of strains with a competitive growth defect in a pooled experiment, a virulence defect in a single-strain assay with an analogous mutation in a different strain background, or in both. Values in the “WT” category represent average filamentation scores of strains with no known virulence defects. Note that most mutations in the WT strain set have not been tested in single-strain virulence assays.

c P-value between strains grown in Lee’s liquid conditions is 6.3 × 10^−6^, in Lee’s solid conditions is 0.028, and between RPMI solid conditions is 6.5 × 10^−6^, as measured by a Students *t*-test.

### Mutant phenotypes are correlated by media state rather than media composition

**Figure 3:**
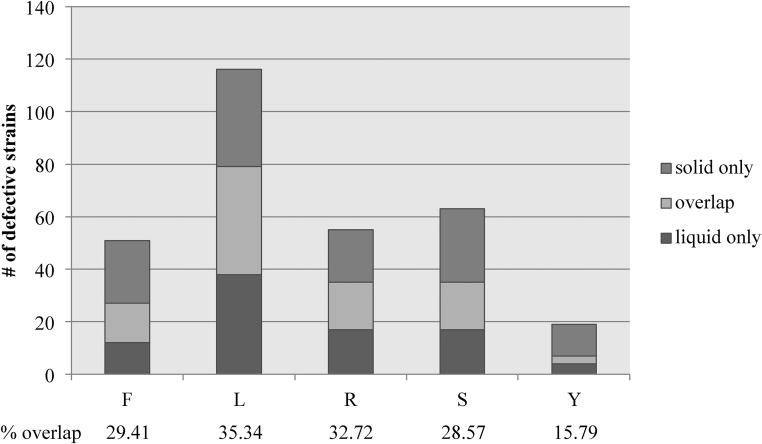
Little overlap exists between liquid and solid filamentation defects. The total number of strains exhibiting a filamentous defect in FBS (F), Lee’s (L), RPMI (R), and Spider (S) liquid and solid media is represented by the total height of the bar for each condition. The number of strains showing defects in both liquid and solid versions of the same media is represented by the light colored section of each bar. The number of strains with solid only (medium gray) and liquid only (dark gray) are also represented on each bar. The percentage of strains exhibiting a defect in both conditions compared to the total number or strains exhibiting a defect in at least one condition is shown below each bar.

We hypothesized that mutant strains would exhibit consistent phenotypic defects between liquid and solid versions (distinct states) of the same media but differ from media with distinct compositions (distinct compositions). However, this hypothesis was strongly refuted by our assay. Overlapping phenotypic defects between media with similar composition but in distinct states ranged between 28.5 and 35.3% for filamentous conditions (Figure 3). In noninducing conditions, where a phenotypic defect represented hyper-filamentation, only three strains had overlapping hyper-filamentous phenotypes in liquid and solid media. These data suggest that there are large differences between filamentation in liquid and solid media even when the media components are largely identical.

**Figure 4:**
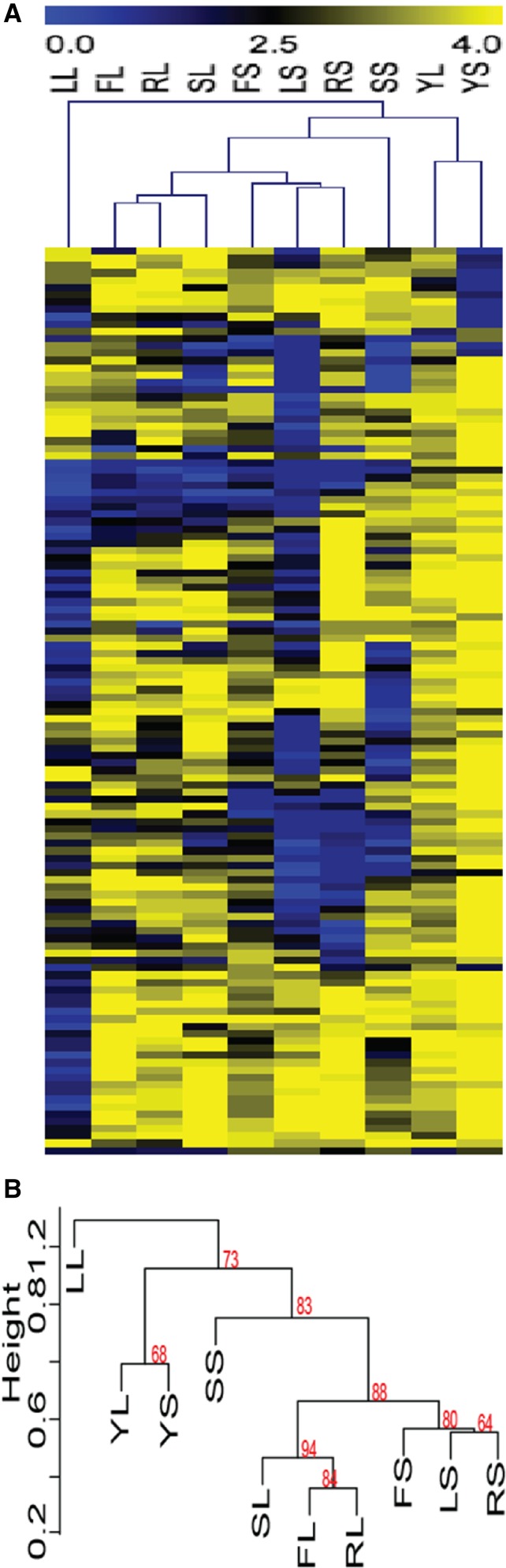
Clustering analysis of filamentation data shows a difference between filamentation in solid and liquid conditions. Hierarchical clustering analysis of the data in Figure 2 and Table S3 identified conditions with similar suites of mutant strains that exhibit filamentation defects. (A). A heat map showing relatedness of phenotypes between strains in each condition. Blue represents strains with phenotypic defects and yellow represents strains with phenotypes close to wild type in each condition. Conditions labels, across the top of the heat map, are the same as those used in Figure 2. Mutant strain phenotype of each of the 124 mutant strains tested is shown across each row. (B). The dendrogram of the hierarchical phenotypic clustering. Approximately unbiased (AU) P-values for each cluster are shown in red.

The lack of phenotypic correlation between the liquid and solid versions of the same media was surprising, and led us to investigate whether any correlations might exist between different media conditions. Clustering analysis was used to compare the conditions tested in our assay. Not surprisingly, noninducing conditions clustered together based on the large number of strains without a phenotypic defect in these conditions (Figure 4). Also clustering together were several inducing conditions that varied in media composition, but that were similar in media state (Figure 4). Liquid RPMI, FBS, and Spider conditions clustered together, and solid RPMI, Lee’s, and FBS were in a distinct cluster. Liquid Lee’s media was an outlier, likely due to the significant number of strains exhibiting a defect in this condition. Solid Spider was also an outlier to these clusters, perhaps due to the distinct scoring mechanism that had to be applied to this condition. These clustering data suggest that physical cues have a stronger impact on the induction and maintenance of filamentation than distinctive nutrient cues, and that the genetic requirements for filamentation in liquid conditions may be quite unique from the requirements for filamentation in solid conditions.

### Transcriptional clustering analysis confirms solid/liquid divide

Based on the variations we observed in our phenotypic analysis, we hypothesized that gene expression would vary greatly between conditions and that identifying factors regulated in common between conditions might allow for the elucidation of media-independent filamentation response genes. These assays would also provide novel insight into the expression profiles of *C. albicans* cells grown on agar plates, which, to our knowledge, have not been attempted previously. RNA was extracted from cells grown for 3 hr in liquid or solid filamentation media at 37° or in noninducing media at 30° and analyzed by RNAseq in triplicate for all experimental and control conditions. As expected, variability was observed in genes differentially regulated in the filamentation induction conditions, although overlap between conditions was noted (Table 3 and Table S4).

**Table 3:** Percent overlap of genes upregulated in distinct filamentation conditions

Conditions	FL	FS	LL	LS	RL	RS	SL	SS
FL	100.00							
FS	42.16	100.00						
LL	56.27	40.05	100.00					
LS	37.83	50.47	32.99	100.00				
RL	44.19	35.12	41.40	36.21	100.00			
RS	32.56	41.23	26.56	54.12	41.06	100.00		
SL	75.95	44.96	58.11	40.86	42.93	34.98	100.00	
SS	47.02	40.50	36.94	42.53	31.34	34.81	54.77	100.00

Percentage reflects the number of upregulated genes that overlapped between the respective conditions divided by the total number of genes upregulated in both conditions. The conditions are labeled following the convention used in the paper. Shading of cells represents a grayscale heatmap, from low overlap (white) to high overlap (dark gray).

**Figure 5:**
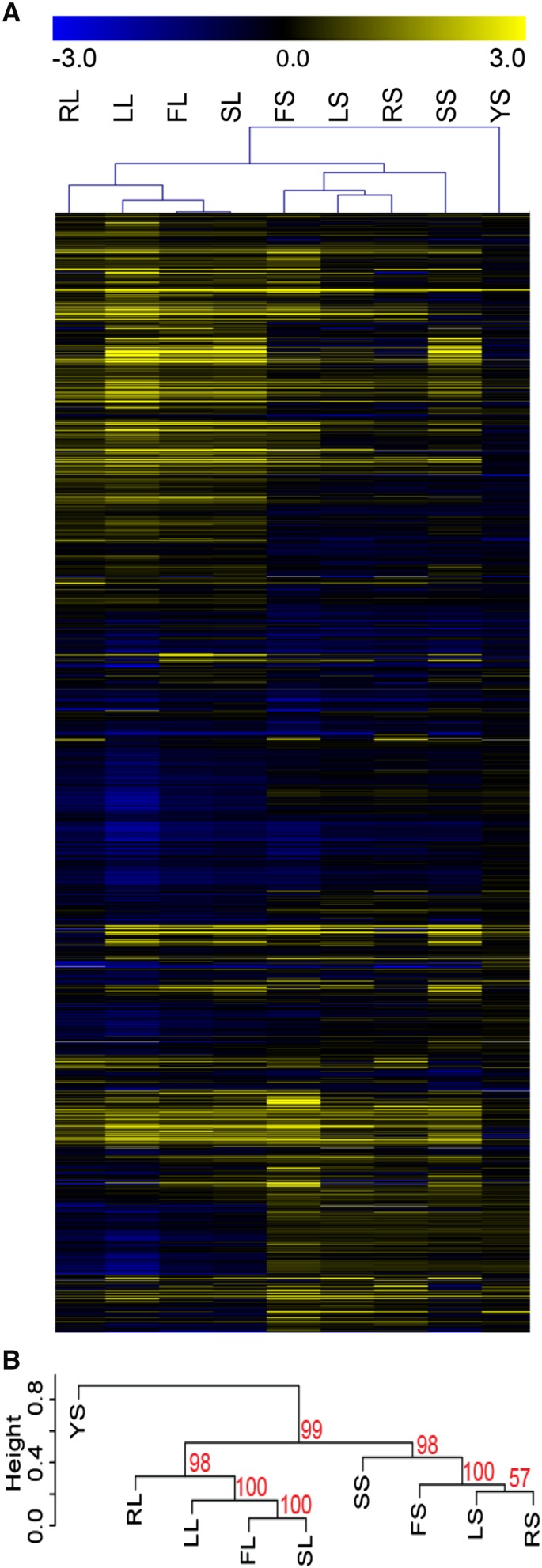
Clustering analysis of gene expression data shows a liquid/solid divide in gene expression. Hierarchical clustering was used to compare the expression of genes in distinct filamentation and control conditions. (A). Clustering revealed related gene regulation between conditions, as shown by the tree at the top of the heatmap. Gene expression was log_2_ transformed prior to clustering. Full details of the expression study are in Table S4. (B). A dendrogram of the hierarchical expression clustering. Approximately unbiased (AU) P-values for each cluster are shown in red.

Clustering analysis was used to identify filamentation conditions with similar expression profiles. Cells grown in solid RPMI, Lee’s, Spider, or FBS media formed one cluster, whereas cells grown in liquid RPMI, Lee’s, Spider, or FBS formed a second cluster, and cells grown in solid YPD media formed an outgroup (Figure 5). While the P-values in the clustering data in the phenotypic assay were moderately supportive of a divide between liquid and solid filamentation programs, the P-values expression data were highly supportive of this divide (Figure 4B and Figure 5B). The combined data strongly supports our hypothesis that physical stimuli drive different patterns of response in *C. albicans* cells induced to form hyphae.

### A conserved filamentation response

**Figure 6:**
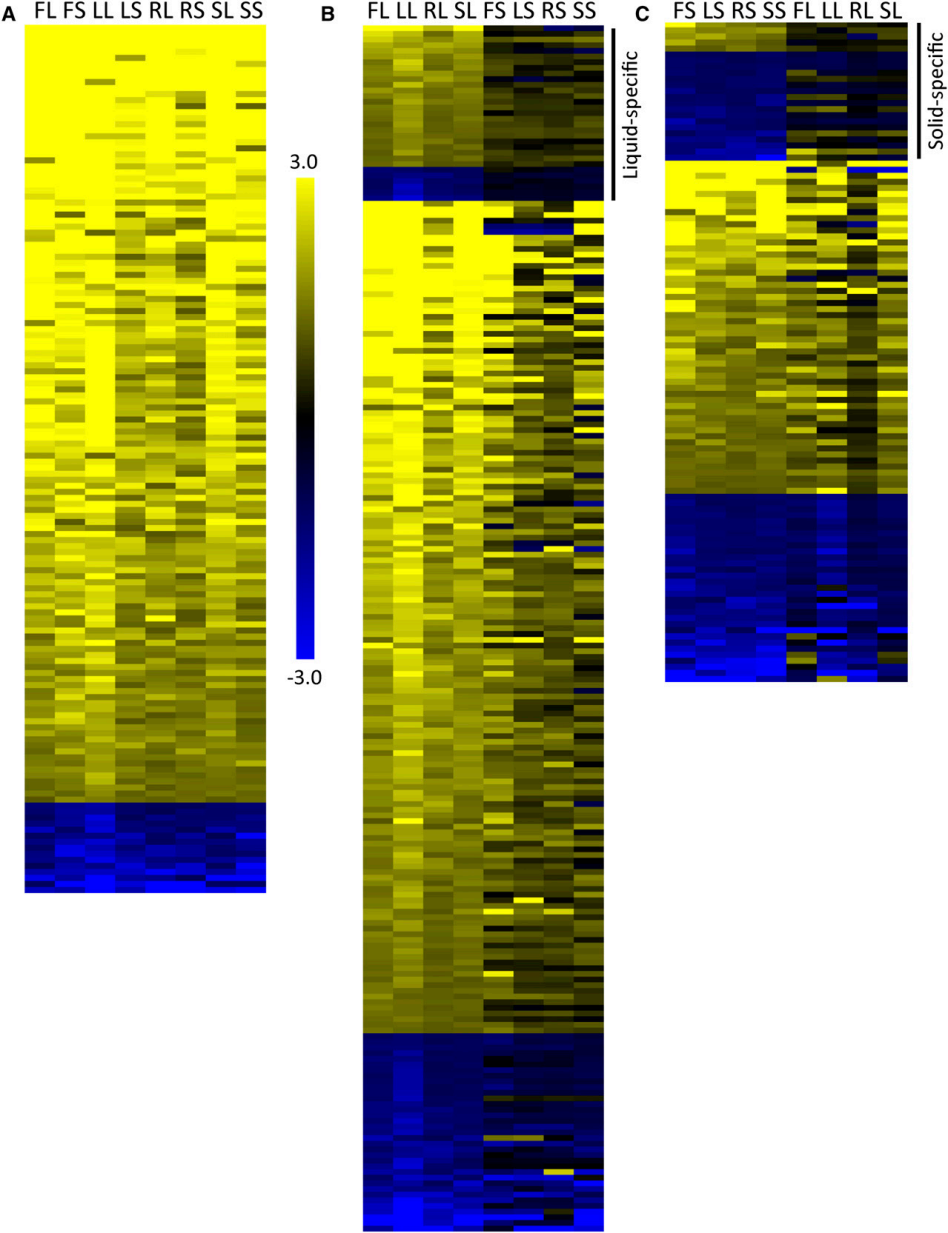
Gene expression analysis in filamentous conditions identifies three patterns of gene expression. Gene expression was measured by RNAseq analysis in each inducing and noninducing condition in triplicate with the YPD liquid used as the normalizing condition. The heat maps represent the average expression of genes in each condition, using the same labels shown in Figure 1. Expression in each condition was normalized by comparing FPKM values for each gene to the FPKM value of that gene in YPD liquid conditions. All data shown have been log_2_ transformed. (A) One hundred forty-four genes showed similar regulation patterns, with 129 genes upregulated and 15 genes downregulated, across all conditions. (B) Three hundred fifty-seven genes showed similar regulation patterns across all liquid conditions. The genes shown are those with liquid-specific profiles, indicated by the bar on the right, and genes with similar expression patterns in at least one solid condition. (C) Two hundred fifty-three genes showed similar regulation patterns across all solid conditions. The genes shown are those with solid-specific profiles, indicated by the bar on the right, and genes with similar expression patterns in at least one liquid condition. The genes similarly regulated in all conditions were not included in B and C. Gene identifications and expression levels are shown in Table S5. Data for the full set can be found in Table S2.

Based on the clustering analysis, we identified three core filamentation responses: genes differentially regulated in all filamentation conditions, in all solid conditions, or in all liquid conditions (Figure 6 and Table S5). In total, 2327 genes were upregulated or downregulated twofold or more in at least one condition, representing almost 40% of the *C. albicans* genome. However, only 129 genes were upregulated and 15 genes were downregulated in all filamentation conditions (Figure 6A). Genes encoding cell wall/membrane proteins (*PHR1*, *PGA7*, *PGA13*, *RBT1*, *RBT5*, *HYR1*, *HWP1*, *SAP10*, *IHD1*), adhesins (*ALS3*, *IFF4*, *C1_13100W*), alcohol dehydrogenases (*ADH1*, *ADH5*, *C6_04410C*, *ADH2*), transcription factors (*TRY6*, *ZCF26*, *WOR3*, *ZCF38*, *BRG1*, *HAC1*), and iron uptake and utilization genes (*ALS3*, *CFL2*, *CFL11*, *FET34*, *FRE9*, *FRP1*, *FRP2*, *FTH1*, *RBT5*, *SIT1*) were among the genes upregulated in common between conditions.

It is difficult to compare assays between research groups due to the variation in experimental conditions, controls used, and times tested. Our controls were at a lower temperature, and it is possible that some of the genes differentially regulated in common between the inducing conditions were regulated in response to growth at 37° rather than filamentation. However, a number of the genes upregulated in all conditions in this study have been shown by others to be upregulated in certain filamentation conditions. Notably, *ALS3*, *HWP1*, *RBT1*, *IHD1*, and *DCK1*, identified as part of the core filamentation response to three distinct liquid filamentation conditions at 3 hr of induction by Martin *et al.* 2013 were also upregulated in all filamentation conditions in our assay. Three genes from the Martin *et al.* set, however, were not present in our set: *ECE1* was highly upregulated in all of our filamentation conditions with the exception of liquid Lee’s media while *SUN41* and *HGT2* were only upregulated in a few conditions. Some of the variation we observe between our study and the Martin *et al.* study may be due to differences in our control conditions and others may have arisen due to the distinct media tested in our assays.

### Unique filamentation response profiles in liquid and solid conditions

In addition to the conserved filamentation response, we also identified unique responses to liquid and solid conditions. Three hundred and one genes were upregulated and 56 genes downregulated in all liquid conditions. Of these differentially regulated genes, 50 show expression patterns that are completely unique to liquid conditions (not showing similar regulation in *any* solid condition) (Table S5). Many of the liquid-unique genes are not well described, and their expression levels are generally 2-4-fold upregulated within most conditions. There are two exceptions. *HSP21*, which encodes a small heat shock protein that is important for filamentation, virulence, and neutrophil resistance (Mayer *et al.* 2012), is upregulated 2.5-fold to 367-fold (average 119-fold) among the distinct liquid filamentation conditions tested. *C7_00350C*, encoding a protein of unknown function, is upregulated 4.5–17.5-fold in the same conditions.

There was less coordination in genes upregulated in all solid conditions. One hundred eighty-nine genes were upregulated and 64 genes were downregulated in all solid conditions. Few genes, however, were uniquely-specific to solid conditions, with five genes upregulated and 18 genes downregulated only in solid conditions (Table S5). One interesting gene upregulated in only solid conditions was the transcription factor *CUP9*. This transcription factor negatively regulates Sok1, which, in liquid conditions at least, is important for the degradation of Nrg1, a negative regulator of filamentation (Lu *et al.* 2014). The upregulation was not high, between two- and fourfold, but could suggest a distinct role for *CUP9* in cells grown in solid filamentation conditions. Overall, our data suggest that there is a core change in gene expression during filamentation that is independent of induction conditions, and that there are distinct responses that are specific to the physical state of the inducing media (Table S5).

### Gene upregulation does not correlate with phenotypic defects

**Figure 7:**
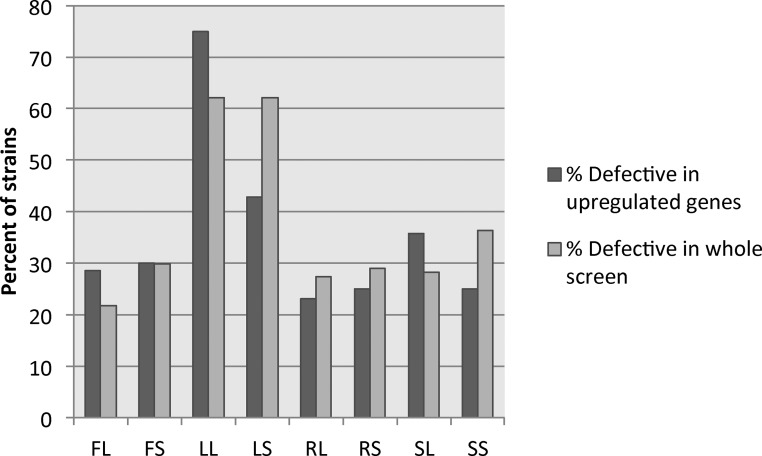
Gene upregulation is not linked to phenotypic defects in coordinate conditions. The expression of genes represented in the mutant collection were compared to the mutant phenotype of the respective deletion strain in each condition. For each condition, the number of strains with a severe phenotypic defect and upregulation of the respective gene were compared to the total number of strains exhibiting a defect in that condition to calculate the percentage defective in upregulated genes (dark gray bars). This is compared to the percentage of strains showing a phenotypic defect in each condition (light gray bars).

Studies have shown that upregulation of genes in environmental conditions is not predictive of the phenotype of strains bearing mutations in those genes in the same environmental conditions, although this is a common prediction (Winzeler and Davis 1997; Giaever *et al.* 2002). Observations in *C. albicans* filamentous cells suggest that upregulation of genes in single-condition assays is also not predictive of phenotype (Banerjee *et al.* 2008; Martin *et al.* 2011), and we wanted to determine whether this held true in our assay as well. We extracted the expression data for genes that were mutated in each of the 124 mutant strains tested for filamentation defects. We first looked at genes from the 129 genes that were heavily upregulated in all conditions. This included *CFL11*, *CIP1*, *BRG1*, *C4_00080C*, *PHR1*, and *DCK1*, which were also tested in our phenotypic screen. From this set of six genes, only *PHR1* showed significant filamentation defects across most inducing conditions (Table 4). When we compare the set of genes showing upregulation in a particular condition and the phenotype of the coordinate mutant strain, the percentage of genes with both upregulation and a severe filamentation defect hovers ∼30% for all assays tested with the exception of Lee’s media (Figure 7, dark bars). The majority of mutant strains exhibiting filamentation defects in each condition, with the exception of Lee’s media, show no coordinate gene upregulation in a wild-type strain. The percentage of mutant strains with defects in a particular condition showing coordinate upregulation of that gene in the same condition largely mirrors the percentage of tested strains showing defects in each condition (Figure 7, light bars), suggesting that overlap of gene function and expression may simply be due to chance. These data support prior observations that overall expression levels are poor predictors of gene importance in particular processes.

**Table 4:** Low filamentation scores do not correlate with gene upregulation

Condition*^a^*	FL		FS		LL		LS		RL		RS		SL		SS	
Gene	exp	fil	exp	fil	exp	fil	exp	fil	exp	fil	exp	fil	exp	fil	exp	fil
PHR1	2.73	2.50	2.28	0.00	2.43	1.83	1.51	0.83	2.70	2.17	2.08	0.00	2.70	2.50	1.52	3.17
BRG1	3.85	1.83	4.94	2.00	3.95	0.67	4.36	0.00	2.74	2.67	3.14	4.00	3.55	2.83	2.74	2.67
DCK1	2.31	3.83	2.60	3.67	2.06	2.50	2.32	4.00	1.62	3.67	1.49	4.00	2.29	4.00	1.56	0.83
C4_00080C	3.12	4.00	2.20	3.17	4.65	1.00	2.30	4.00	2.61	3.33	1.87	3.83	2.88	4.00	2.67	3.00
CFL11	8.40	3.83	8.16	3.83	8.78	1.17	7.18	2.33	8.20	4.00	6.27	4.00	8.10	4.00	5.80	4.00

a Condition name follows the convention used throughout the paper. exp, gene expression (log_2_); fil, filamentation score for strain bearing a mutation in the indicated gene. Filamentation scores of 2.5 and below are highlighted in gray.

## Discussion

Our work has shown that single-media assays are not reliable indicators of filamentation phenotypes across a broad spectrum of conditions. Our data suggest, instead, that distinct conditions have moderately overlapping genetic requirements for filamentation, and the majority of these genetic requirements are condition-specific. This leads to a question of why *C. albicans* has such distinct genetic requirements for, and transcriptional responses to, filamentation in distinct conditions. One possibility is that distinct stress responses converged onto the same phenotypic solution, filamentation. When cells are starved of nutrients, morphogenesis from yeast cells to filamentous cells allows *C. albicans* to forage distal locations from the mother cell in a rapid fashion. When *C. albicans* is ingested by a phagocytic cell, the morphological change from yeast to filamentous form allows the fungus to physically escape the larger phagocytic cell (Lorenz *et al.* 2004). And coordinate with changes in physical shape, filamentation also changes the suite of molecules presented on the surface of the cell, which allows *C. albicans* to evade the immune system, scour nutrients from host cells, and adhere tightly to cell surfaces. Differences in the genetic requirements for filamentation may exist because these responses may have not yet coalesced into a single filamentation pathway.

Another possible reason *C. albicans* has divergent responses to the filamentation induction condition is that distinct programs of filamentation would allow *C. albicans* to tailor its response to specific environmental signals. *C. albicans* resides in a wide variety of niches within the human body with very distinct environmental conditions. Filamentous cells in these niches may have unique characteristics that allow them to survive in that environment. This could explain the unique transcriptomes present in each condition, but still leaves the question of why distinct genetic requirements for filamentation in each condition exist and whether these genes are involved in initiation or maintenance of filamentation in these conditions.

At the outset of our experiments, we hypothesized that genetic requirements for filamentation and transcriptional responses to filamentation would be similar in liquid and solid versions of media with the same components. Our data, however, strongly suggest that filamentation programs are distinct in solid and liquid media, both in the genetic requirements for filamentation and in the transcriptional response to filamentation. Based on these observations, we hypothesize that *C. albicans* must differentiate between filamentation in a free-floating state and on surfaces in the gastrointestinal and genitourinary tracts of the human body and that its ability to respond appropriately to these distinct conditions is important for its survival *in vivo*. Thus, filamentation on solid surfaces *in vitro* might mimic filamentation on epithelial or endothelial surfaces within the body and perhaps within tissues as well, while filamentation in liquid media *in vitro* would mimic filamentation of *C. albicans* in bodily fluids or perhaps within phagocytic cells of the immune system. Our data suggest that we need to treat solid and liquid filamentation as two distinct phenotypes and that defects in each process will have different impacts on pathogenesis.

While there are large differences in the genetic requirements for filamentation between conditions, our data identified a number of core genes important for filamentation across conditions (Table 1). The role that most of these genes play in the initiation or maintenance of filamentation is currently not known, but the function these genes or their orthologs in related species play in other cellular processes may provide hints about their functions. Two genes from this core set of genes with broad roles in filamentation, *STT4* and *TSC11*, may contribute to filamentation via their roles in regulating the actin cytoskeleton. *Saccharomyces cerevisiae* orthologs of Stt4, a phosphatidylinositol 4-kinase, and Tsc11, a member of the TORC2 complex, act in pathways that activate the GTPase Rho1, which regulates the polarization of the actin cytoskeleton and the Pkc1 cell wall integrity pathway in *S. cerevisiae* (Schmidt *et al.* 1997; Levin 2005). Polarization of the actin cytoskeleton is required for the filament formation in *C. albicans* (Akashi *et al.* 1994) (Cali *et al.* 1998) and blocking polarization by either destabilizing actin can prevent expression of hyphal responsive genes (Wolyniak and Sundstrom 2007). It seems likely that these genes are playing similar roles in *C. albicans* and implicates other members of their signaling pathway (Stt4) or complex (Tsc11) in filamentation as well.

Cell wall structure is distinct between filamentous and yeast cells (Lowman *et al.* 2014), and four genes in the core filamentation set, *KRE5*, *PHR1*, *IRE1*, and *C4_04090C*, may play a role in the generation of distinct cell wall features. Both *KRE5*, which encodes a putative glucosyltransferase, and *PHR1*, which encodes a cell surface glycosidase, have roles in altering cell wall glucan composition (Skrzypek *et al.* 2017). Tangentially, *IRE1*, which encodes a protein kinase important for cell wall integrity that may be involved in the ER-related unfolded protein response (Blankenship *et al.* 2010), and *C4_04090C*, which encodes a putative ER chaperone important for glycoprotein folding (Skrzypek *et al.* 2017), may also be involved in either altering cell wall composition (C4_04090C) or responding to changes in the cell wall architecture (Ire1). The importance of these cell wall linked genes to filamentation across a broad spectrum of filamentation conditions suggests that cell wall changes are required for filamentation and blocking these changes by altering filament glucans or glycoproteins could inhibit filamentation, and thus pathogenesis, *in vivo*.

The identification of core genetic requirements, however, does not rule out the possibility that certain *in vitro* conditions have a predictive value for specific *in vivo* phenotypes. In our assays, RPMI solid media defects appear to correlate with competitive survival or virulence defects in murine tail vein injection models of infections. It is possible that filamentation phenotypes in other media can predict virulence at other infection sites, such as the oral or vaginal mucosa or in intra-abdominal infections. Identifying the unique genetic requirements for these responses could identify targeted approaches for treatment.

Our work has identified problems underlying reliance on single-model systems in the investigation of filamentation in the pathogenic fungus *C. albicans*. However, it also highlights the strength of comparing multiple models of the same process. We were able to identify core genetic requirements and gene expression profiles for filamentation only by looking at a suite of *in vitro* filamentation models. This approach also uncovered distinct programs of filamentation in solid and liquid media that were hidden, in part, by our reliance on liquid models of filamentation for expression profiling.

### Conclusions

Similar to investigators in most other organismal systems, the *C. albicans* field relies on *in vitro* models to study *in vivo* biological processes. Further, studies in *C. albicans*, like studies in other systems, utilize a variety of *in vitro* models to examine a single phenotype. We found very little overlap in the genetic requirements for filamentation among the model systems we tested, and transcriptional responses to those conditions also varied widely between models. This suggests that reliance on a single-model system for filamentation will generate both false-positive and false-negative results. It is only with a broad screen across multiple conditions that we can identify genes with filamentation roles conserved across conditions and identify transcriptional responses that are specific to the overall process of filamentation. Discrepancies between model systems are not confined to *C. albicans* filamentation. In many cases where discrepancy exists between results in different model systems, it is generally observed as a weakness of *in vitro* investigations (Haibe-Kains *et al.* 2013). In some cases, comparisons can be used to identify the *in vitro* models that best replicate *in vivo* conditions (Wilding and Bodmer 2014), although this appears to be rare. Rather than looking at the differences between model systems as a weakness, we suggest that comparative approaches can be used to increase the likelihood that the outcome of assays best reflect the core requirements for an *in vivo* phenotype. In addition, the identification of themes underlying differences in model systems, like the divergence between solid and liquid filamentation, can identify biological processes overlooked in single-model approaches.

## Supplementary Material

Supplemental material is available online at www.g3journal.org/lookup/suppl/doi:10.1534/g3.117.300224/-/DC1.
