# Supplementary material for: Filamentation Involves Two Overlapping, but Distinct, Programs of Filamentation in the Pathogenic Fungus Candida albicans
Source: G3 (Bethesda). 2017 Sep 25;7(11):3797–808. doi: 10.1534/g3.117.300224 (PMC5677161; doi:10.1534/g3.117.300224)
Supplement: Supplementary file 5 [file 3797FigureS5.pptx]

## Slide 1
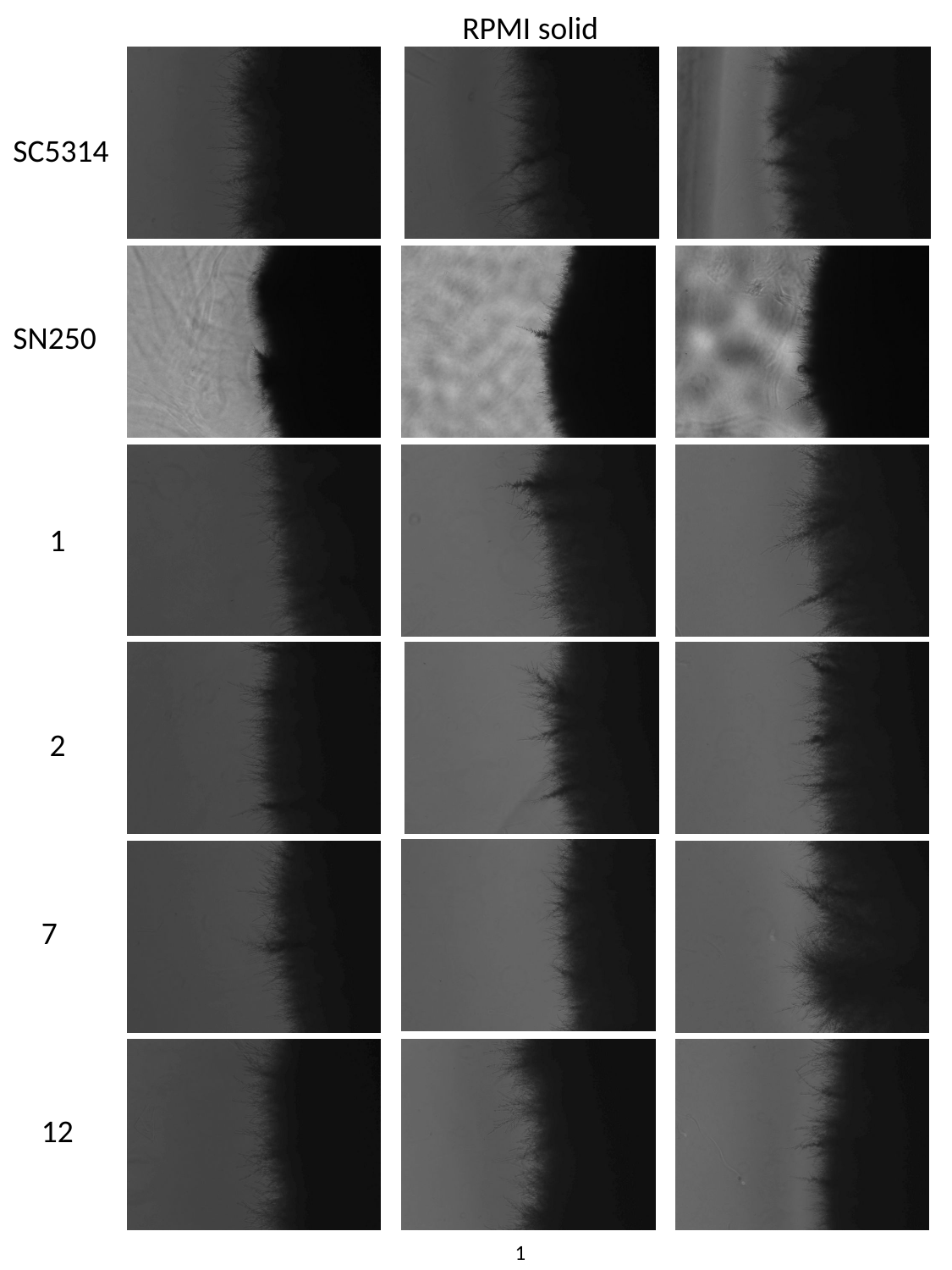

RPMI solid
SC5314
SN250
1
2
7
12
1

## Slide 2
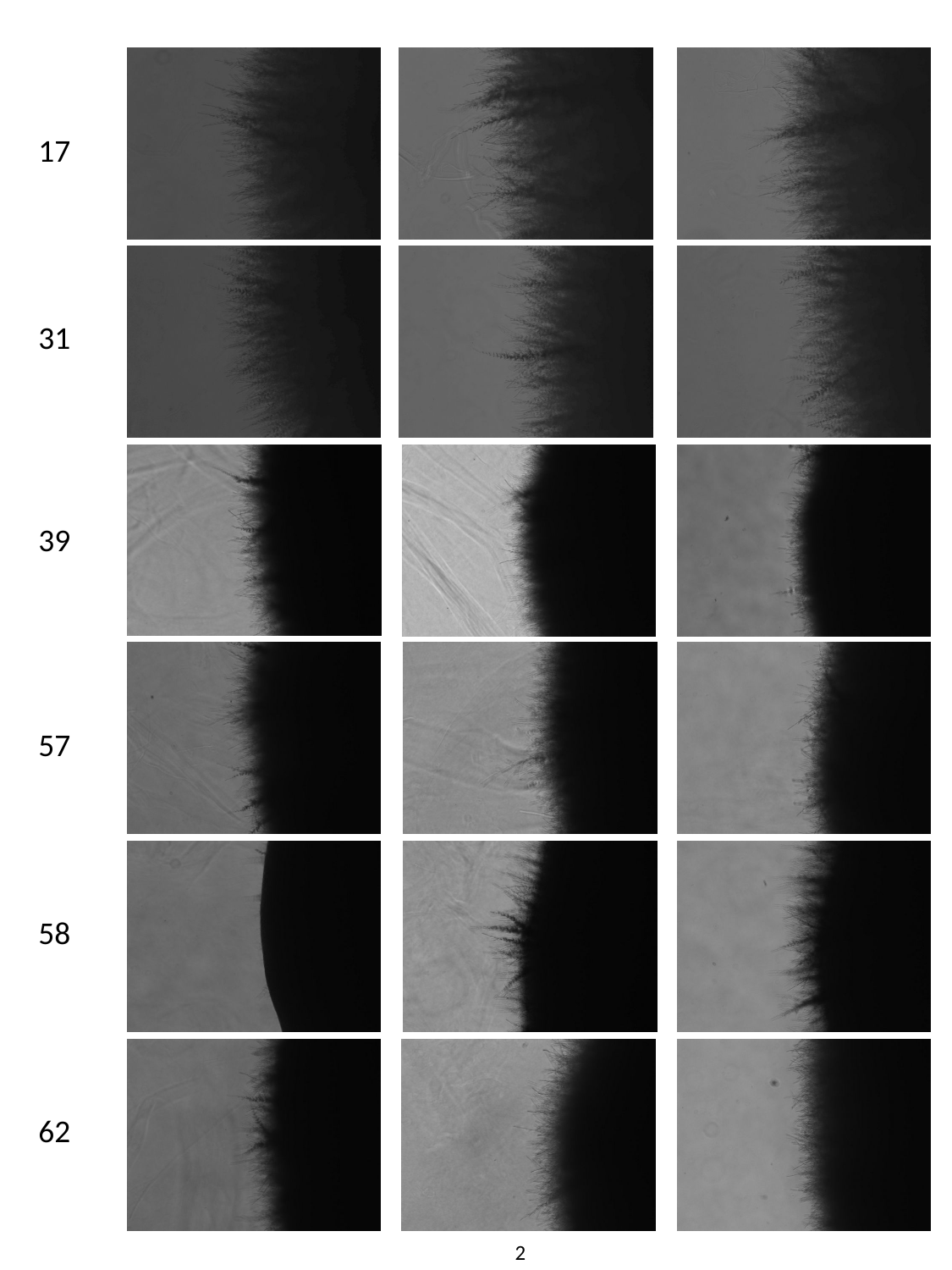

17
31
39
57
58
62
2

## Slide 3
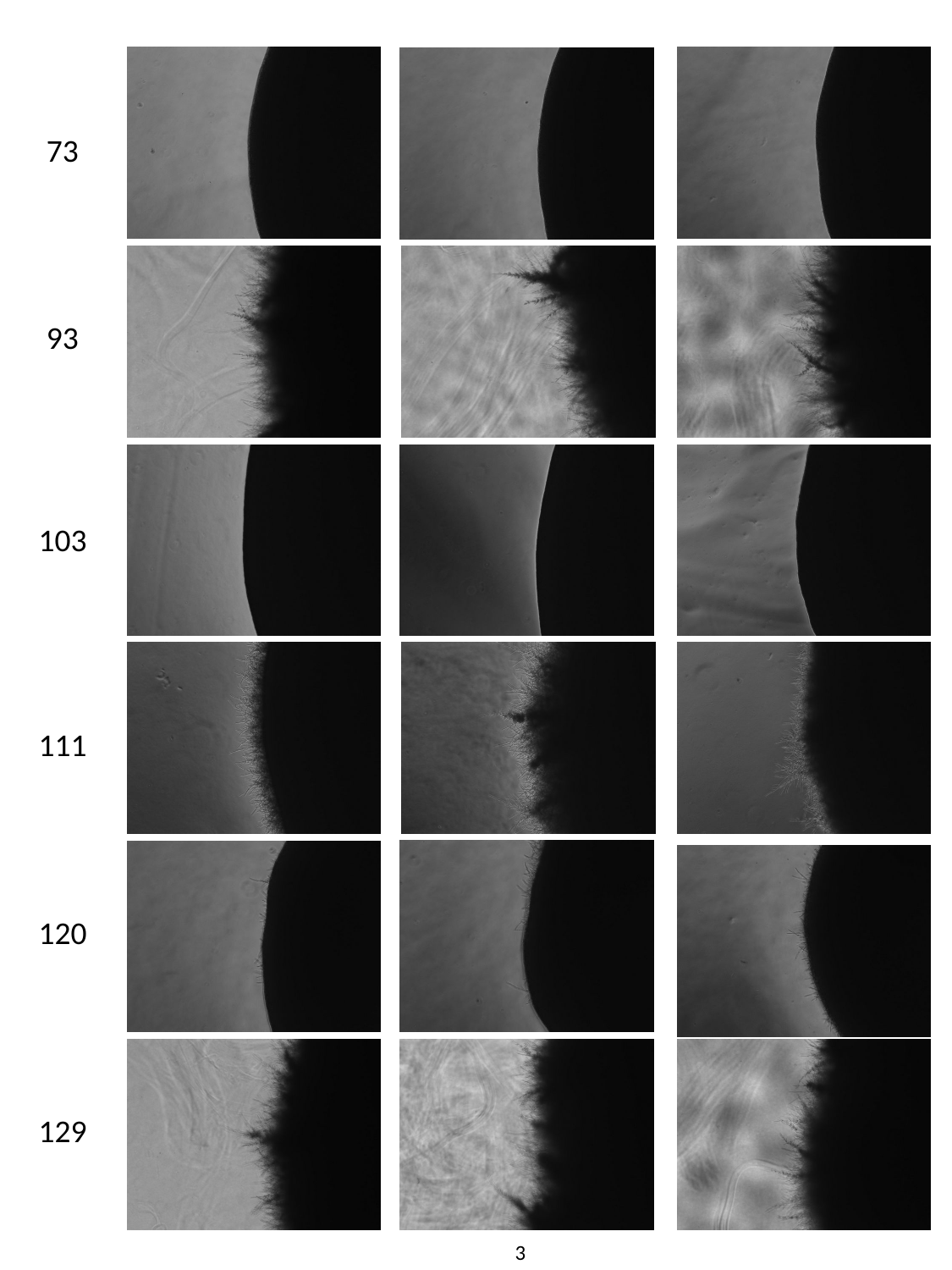

73
93
103
111
120
129
3

## Slide 4
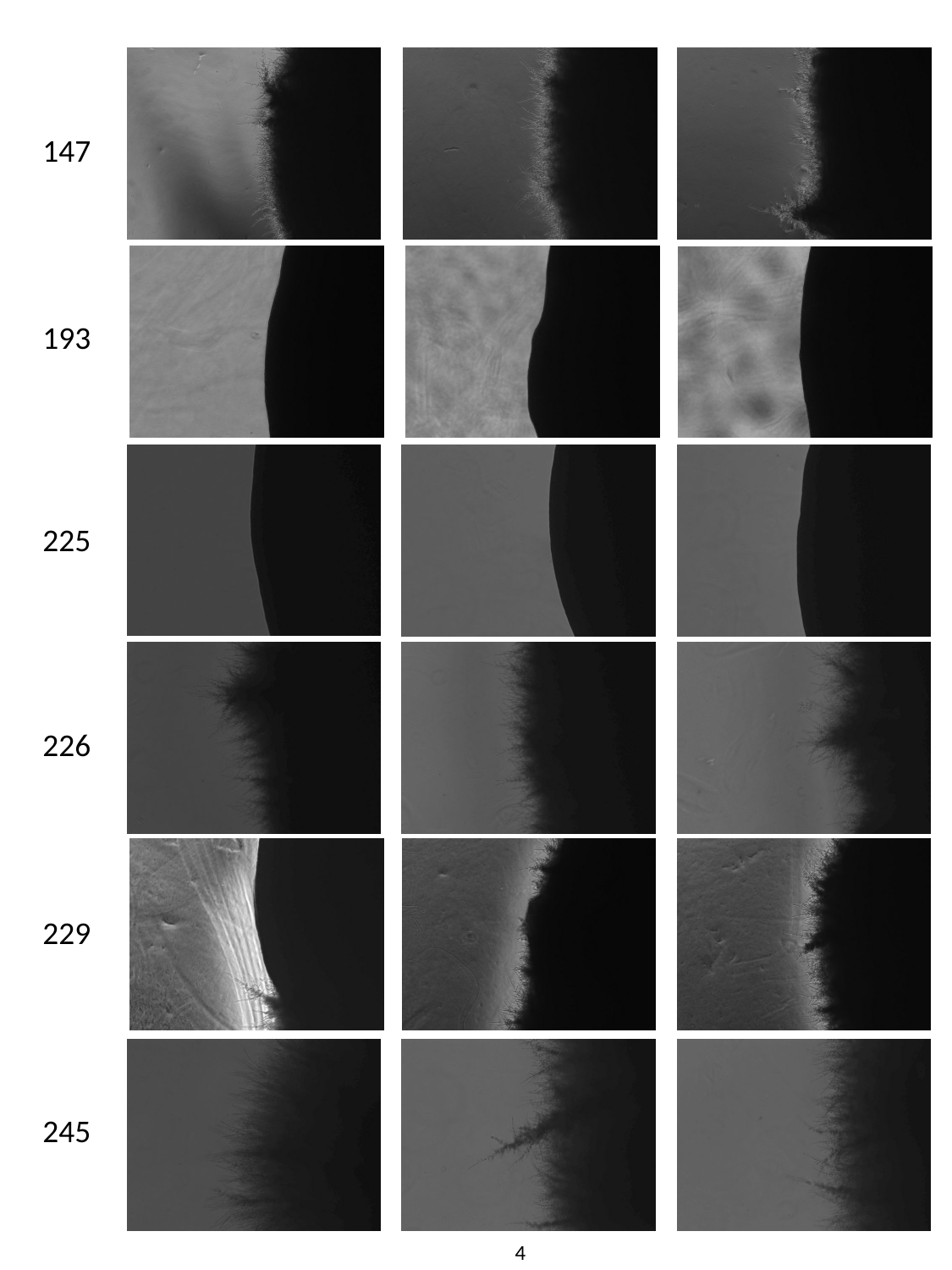

147
193
225
226
229
245
4

## Slide 5
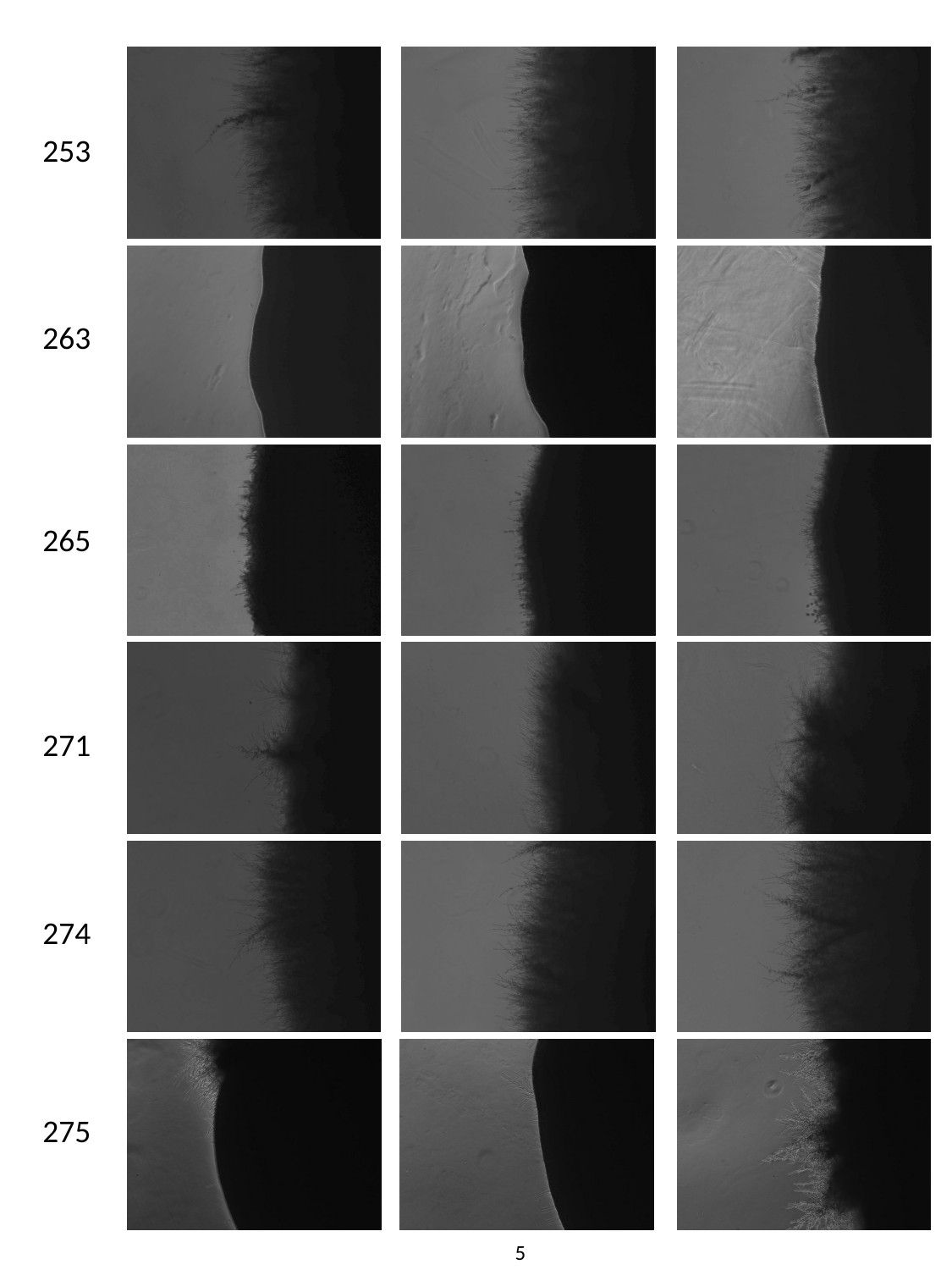

253
263
265
271
274
275
5

## Slide 6
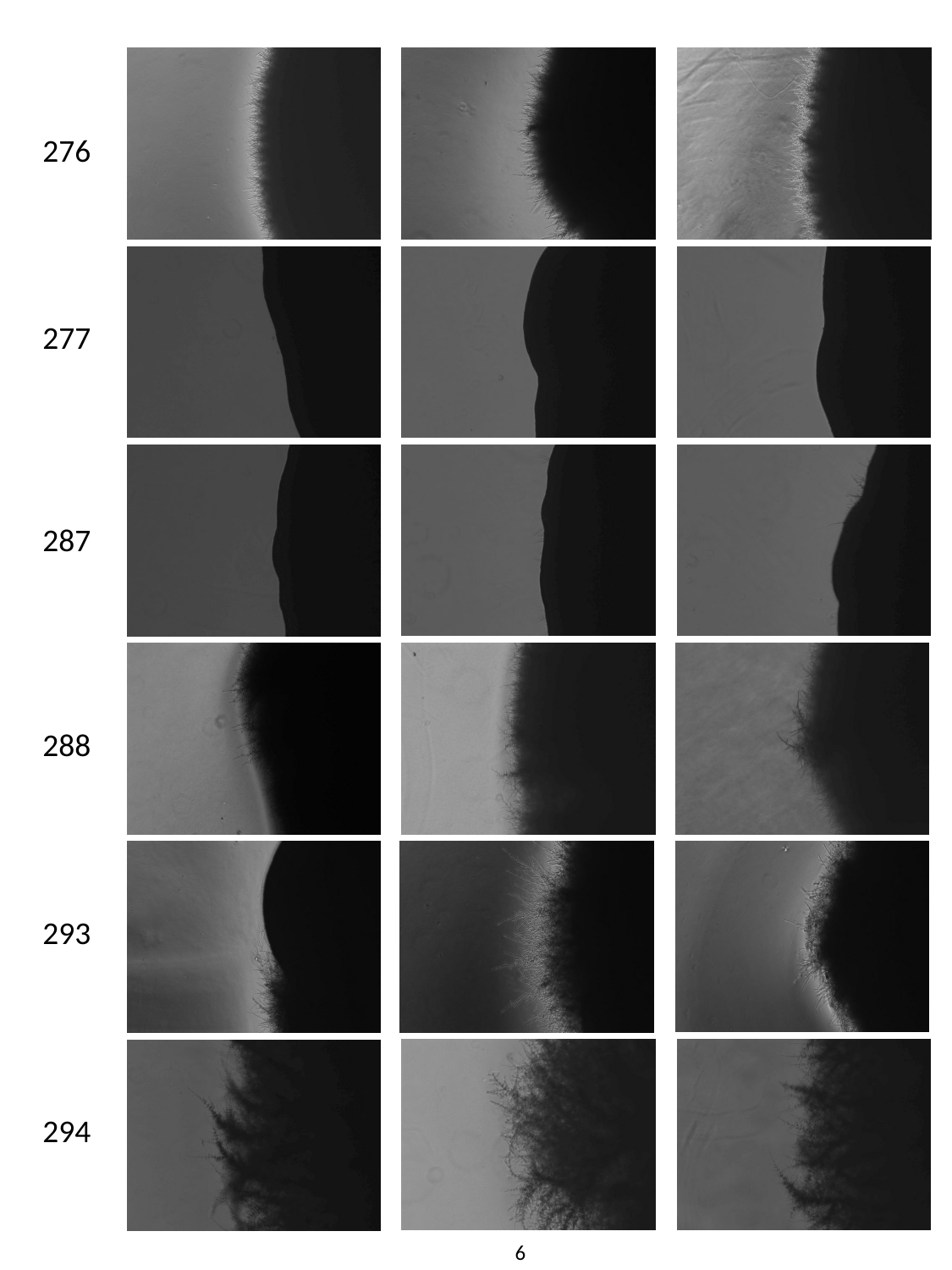

276
277
287
288
293
294
6

## Slide 7
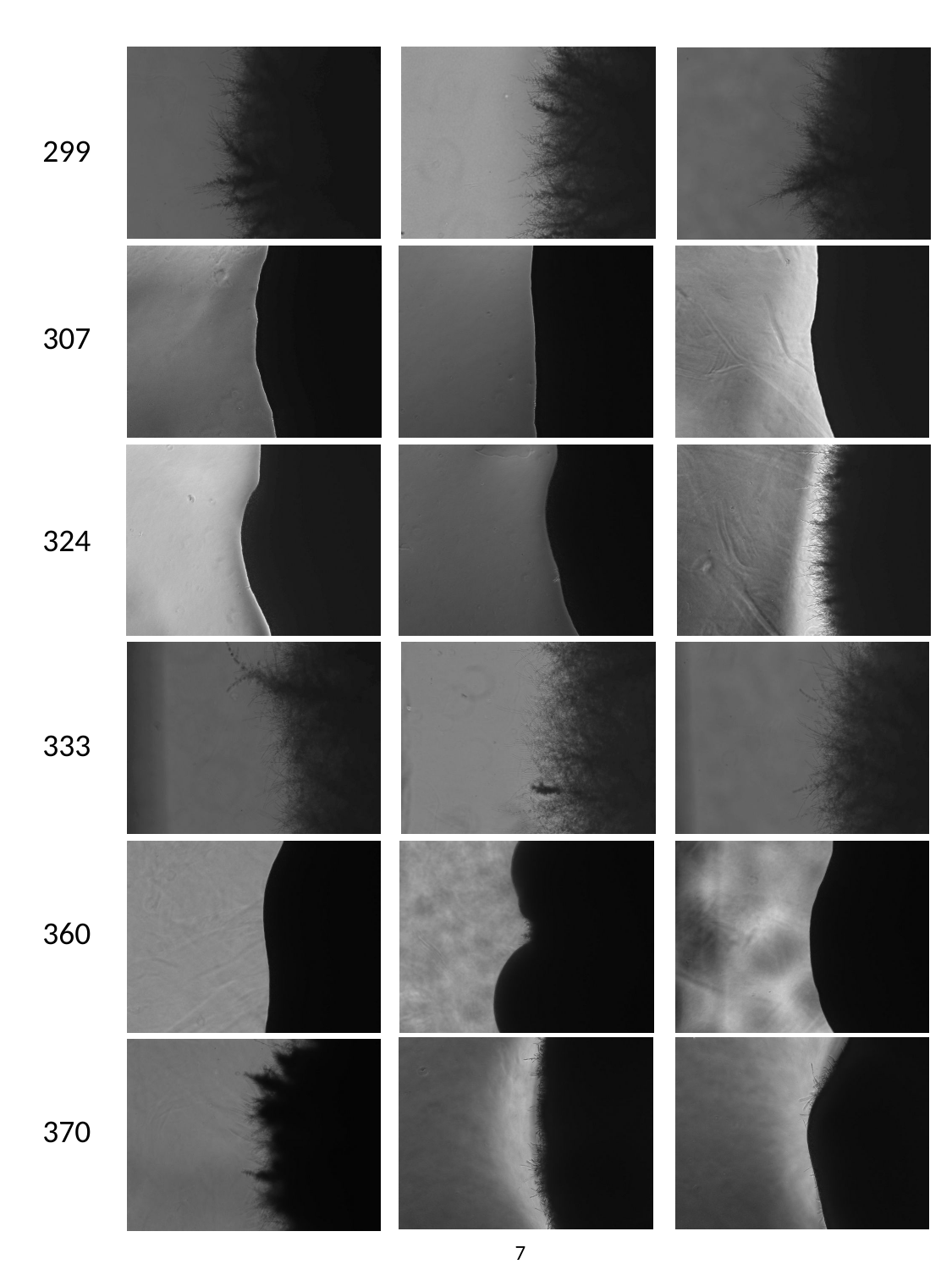

299
307
324
333
360
370
7

## Slide 8
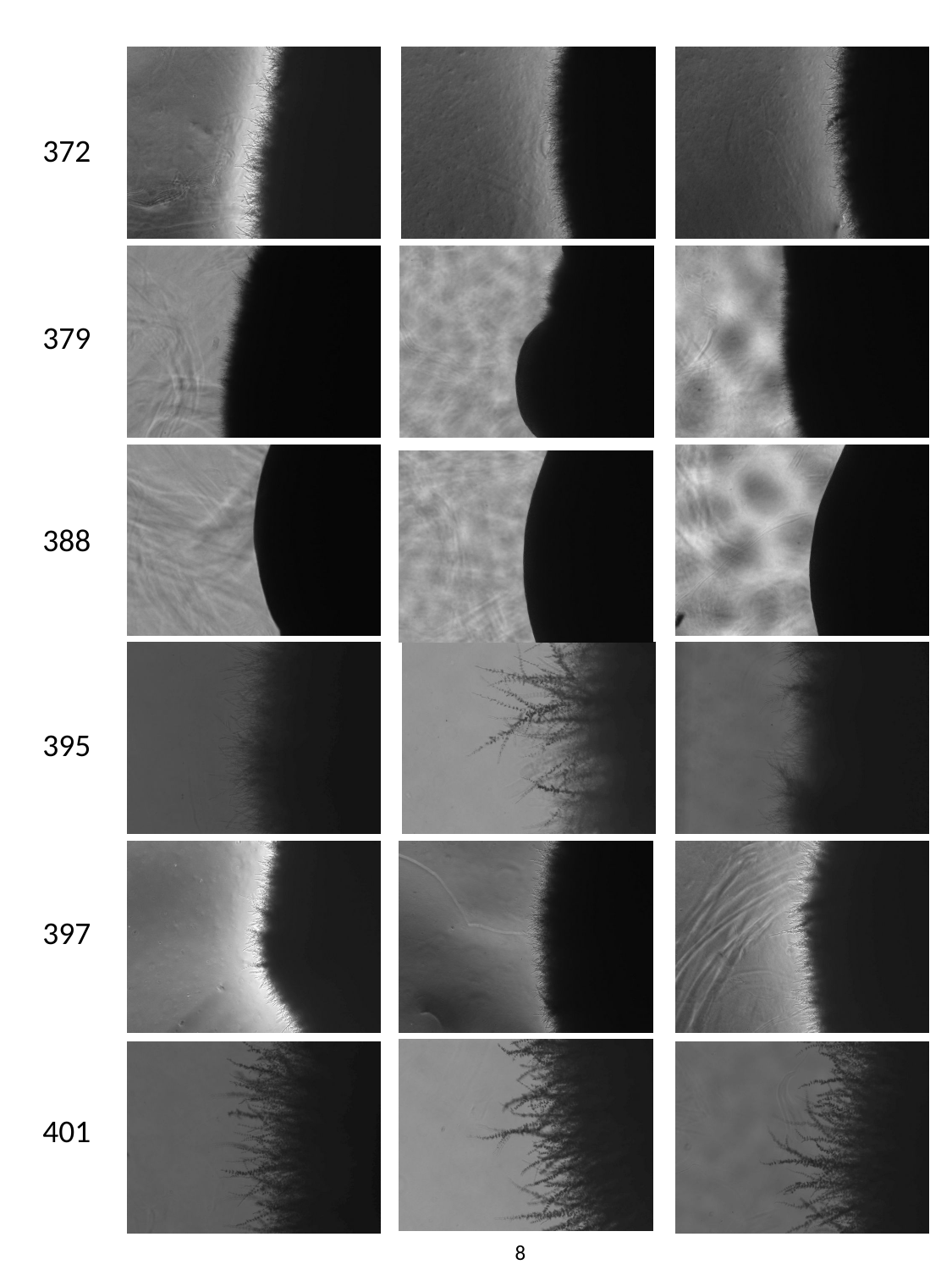

372
379
388
395
397
401
8

## Slide 9
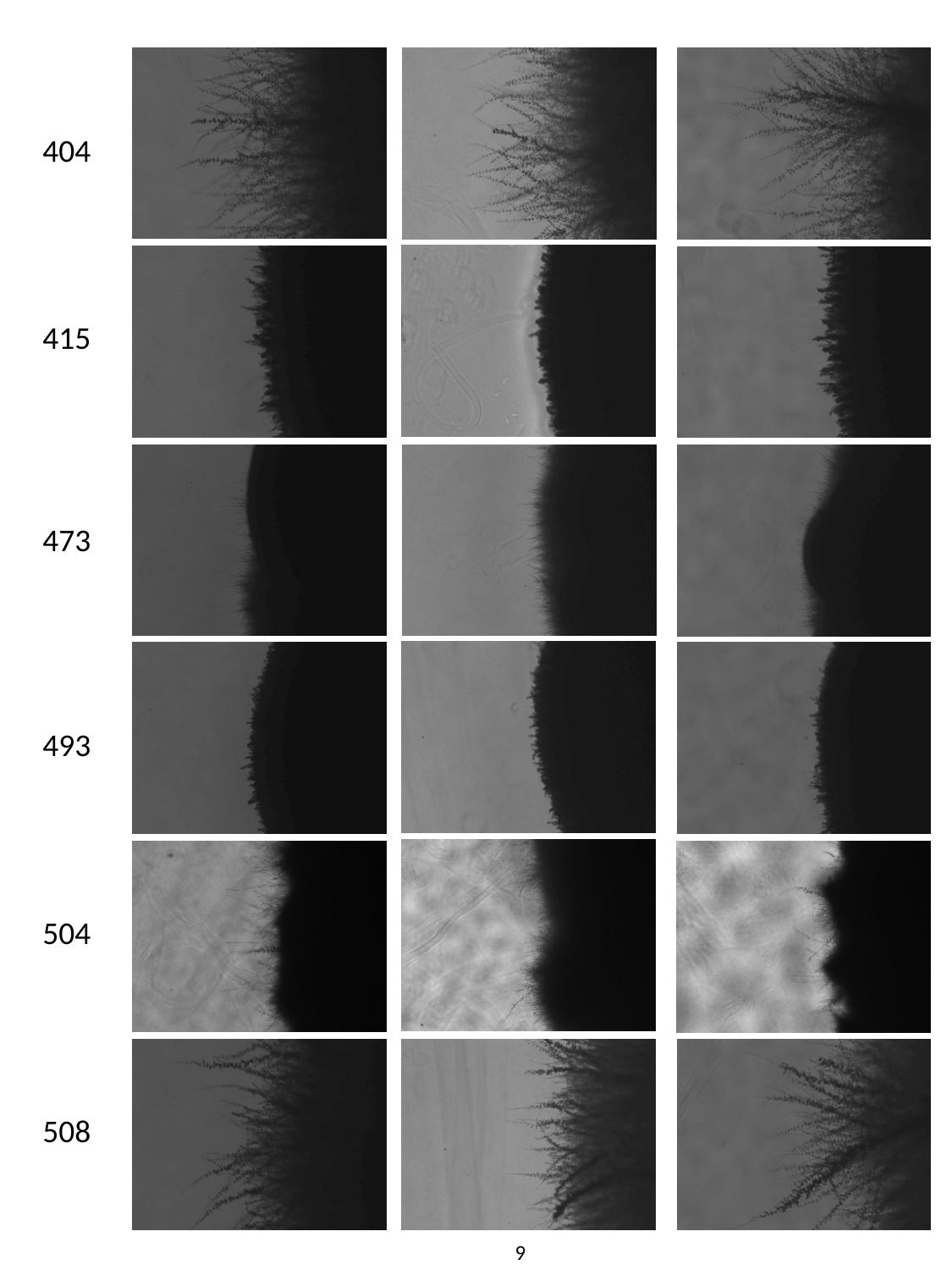

404
415
473
493
504
508
9

## Slide 10
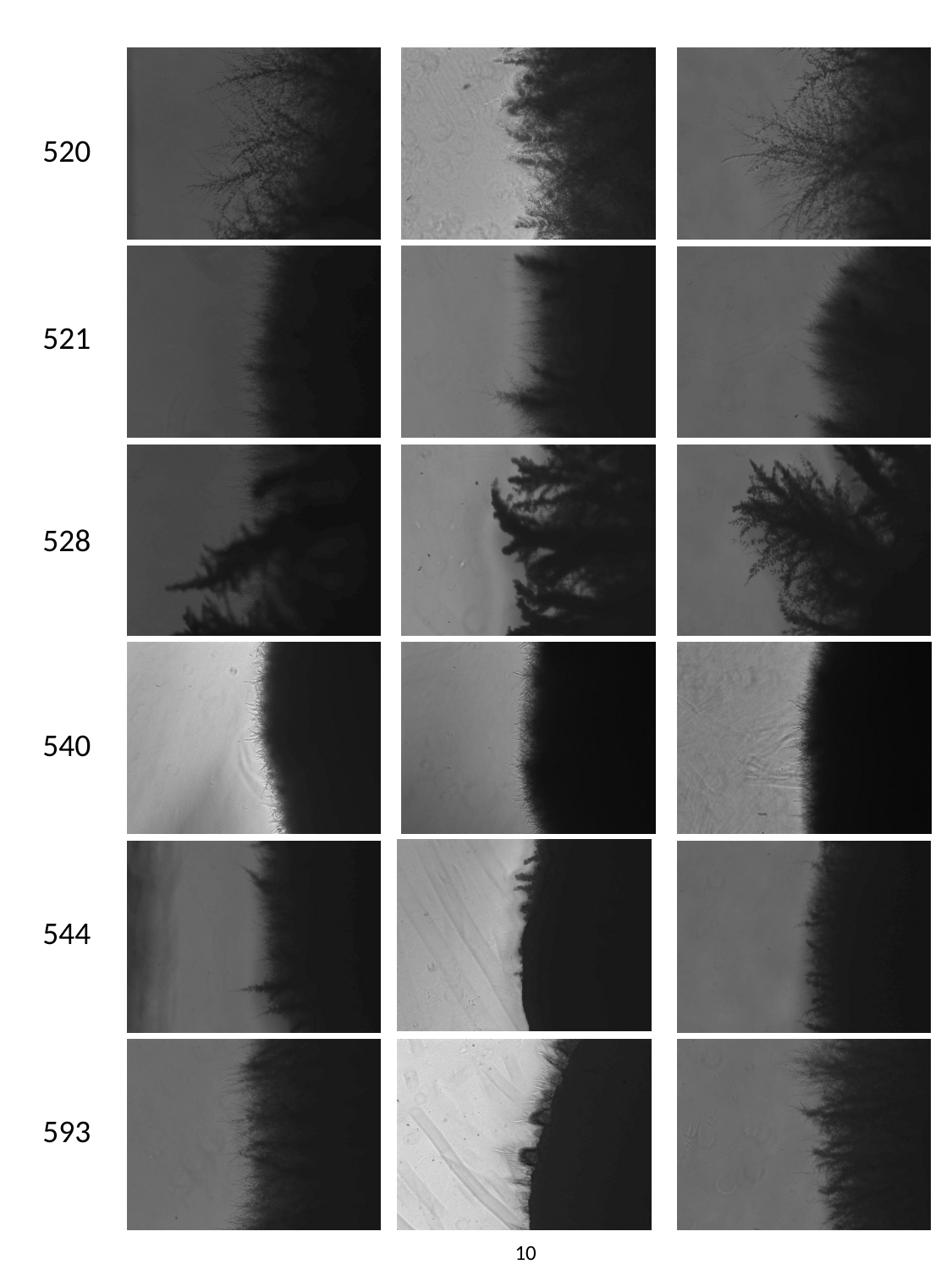

520
521
528
540
544
593
10

## Slide 11
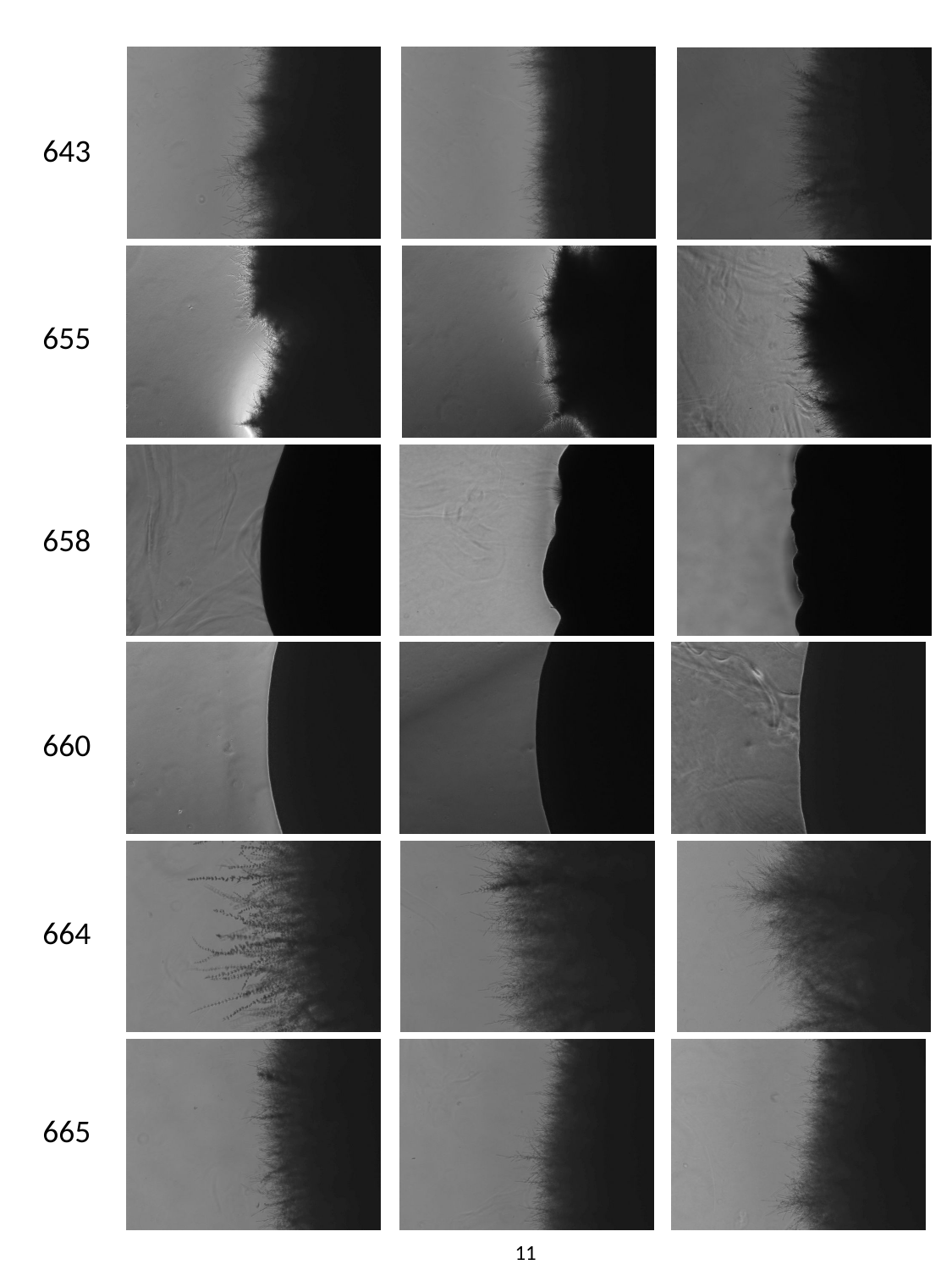

643
655
658
660
664
665
11

## Slide 12
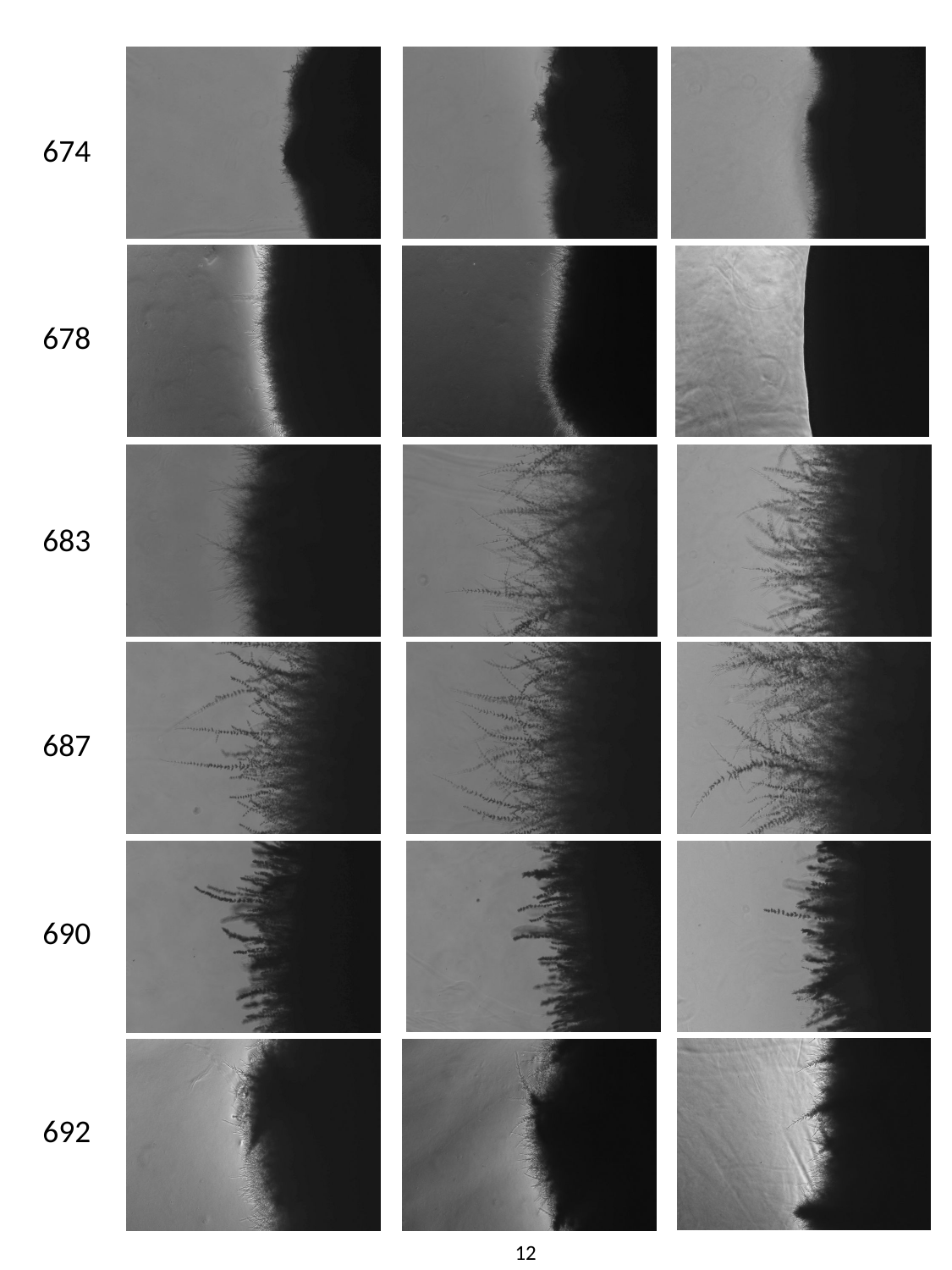

674
678
683
687
690
692
12

## Slide 13
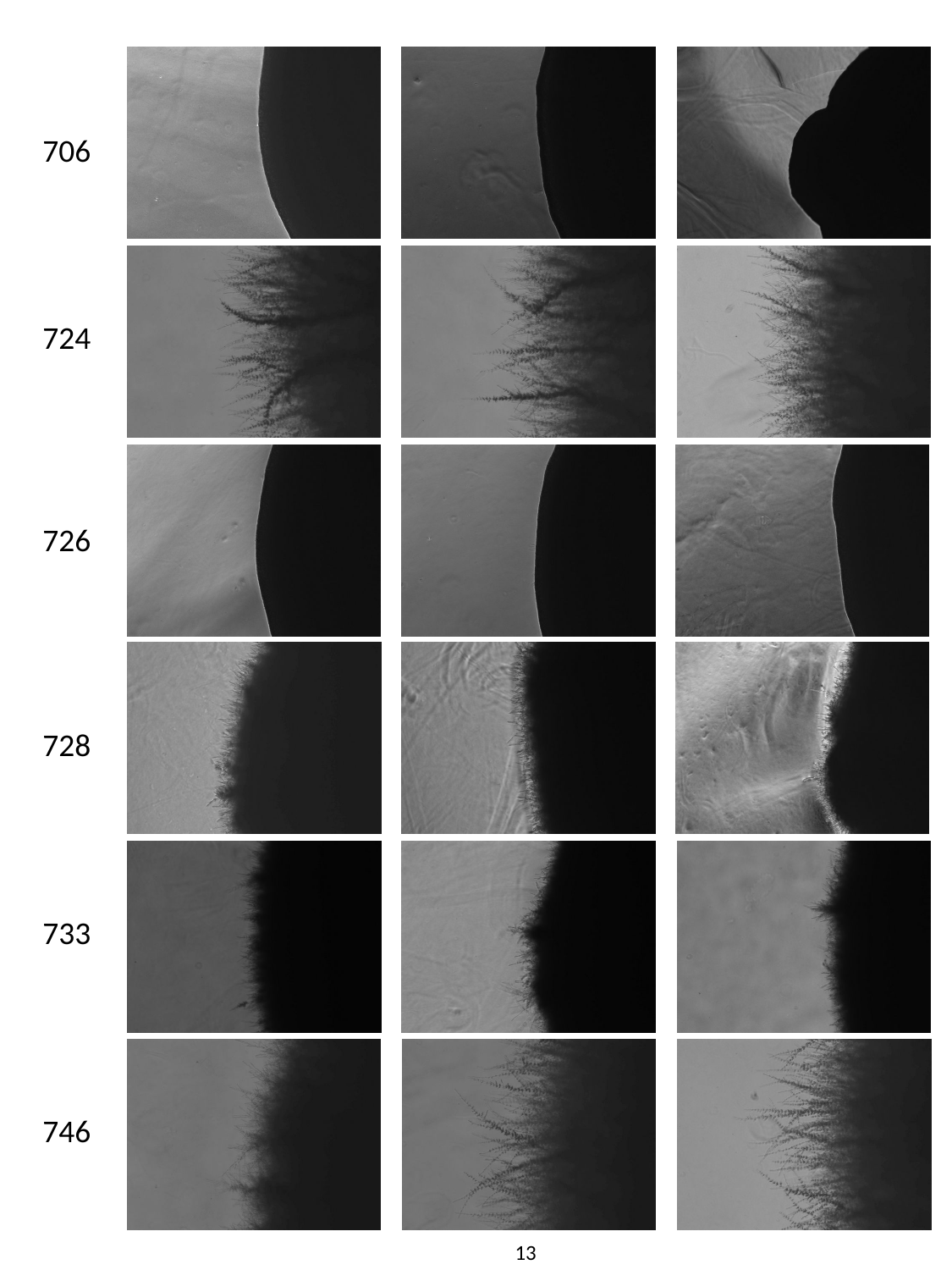

706
724
726
728
733
746
13

## Slide 14
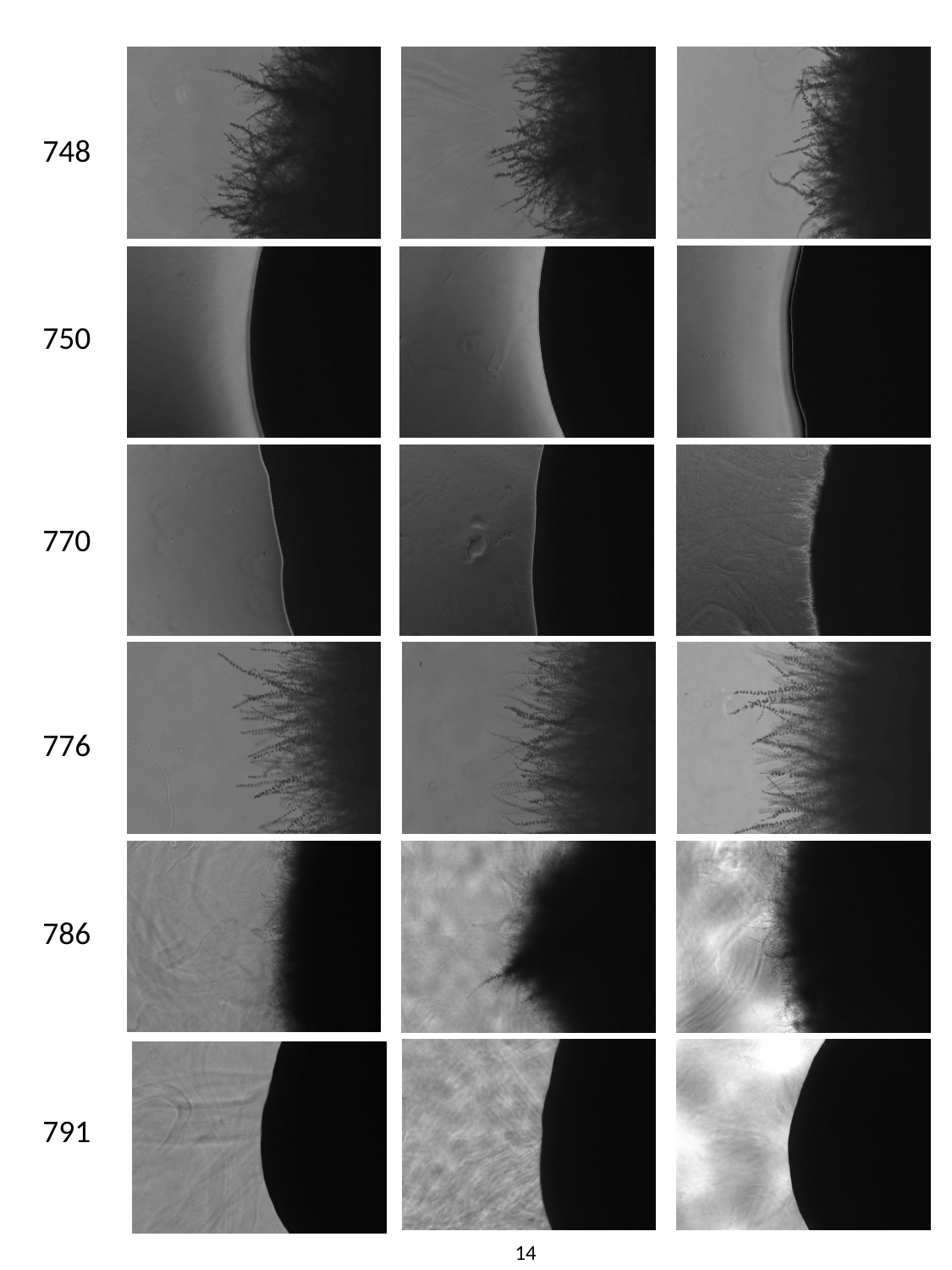

748
750
770
776
786
791
14

## Slide 15
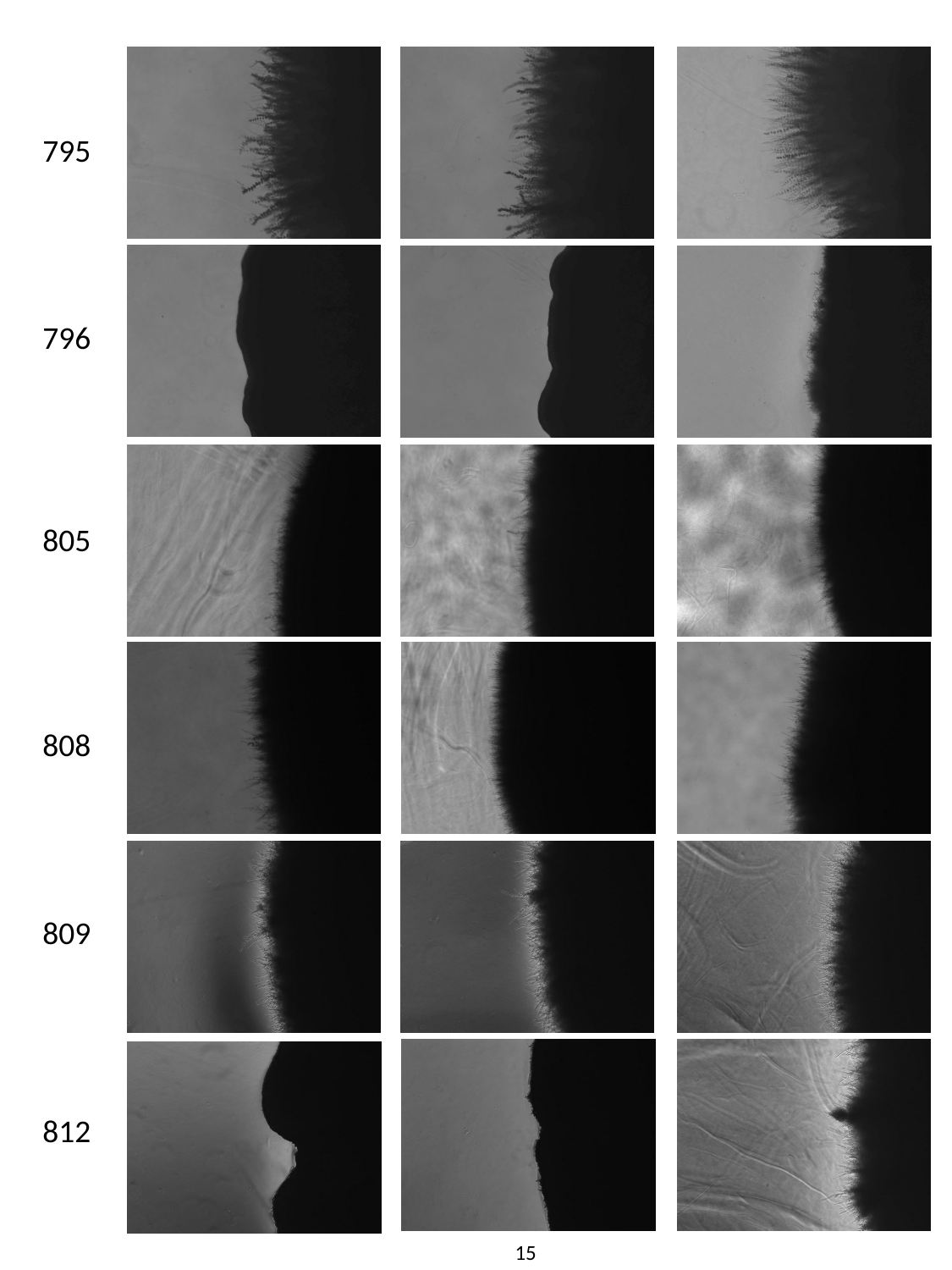

795
796
805
808
809
812
15

## Slide 16
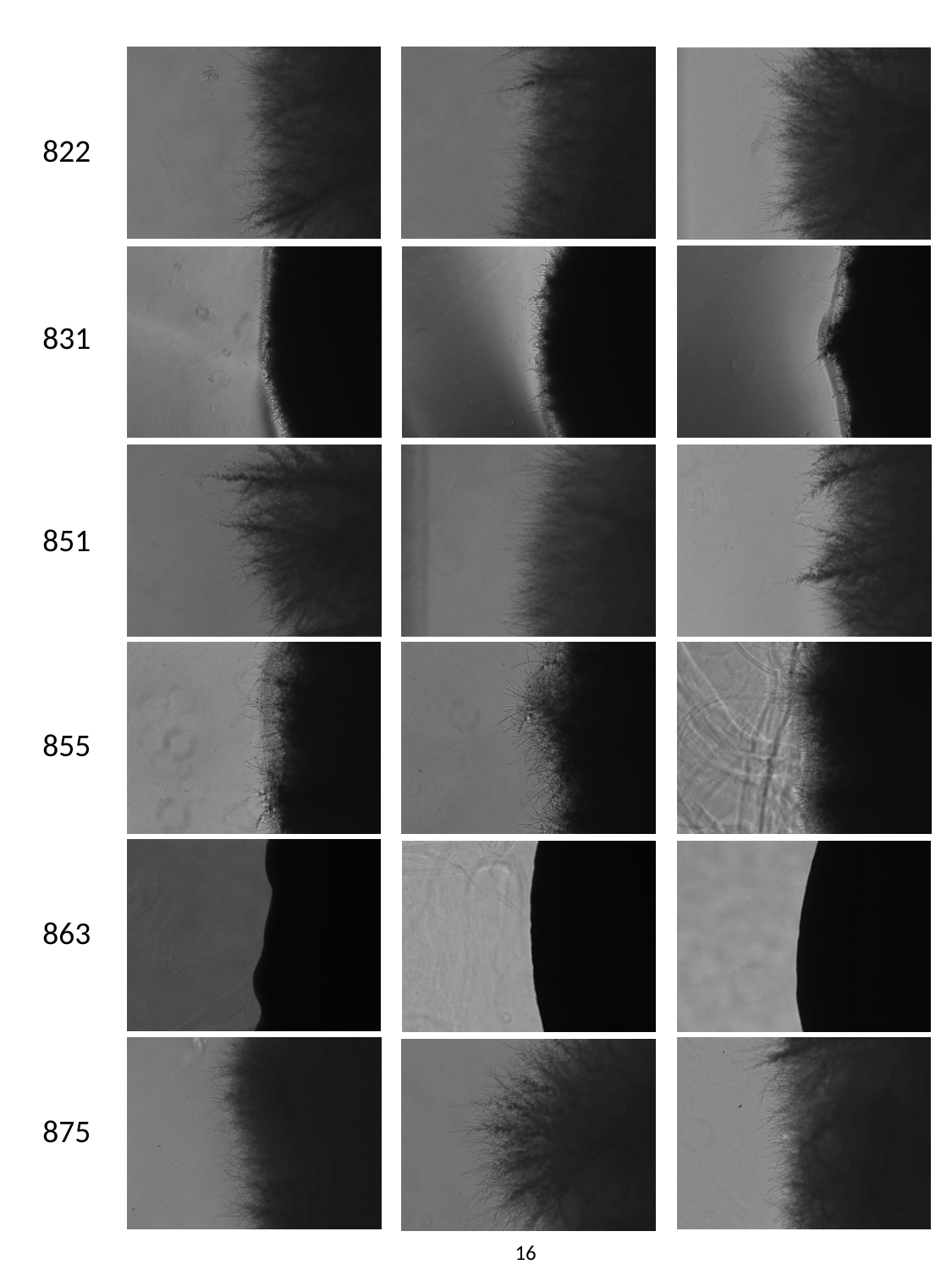

822
831
851
855
863
875
16

## Slide 17
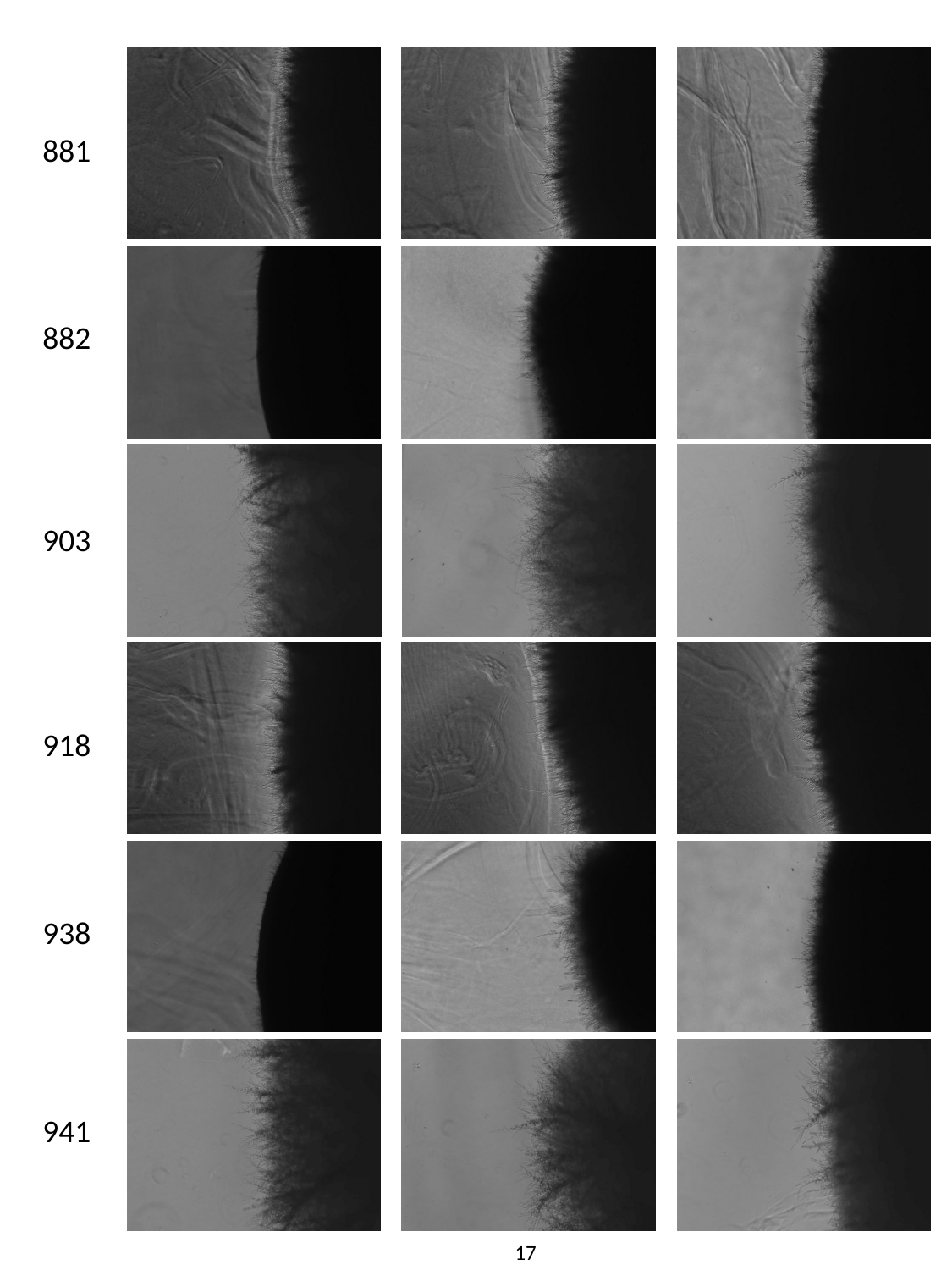

881
882
903
918
938
941
17

## Slide 18
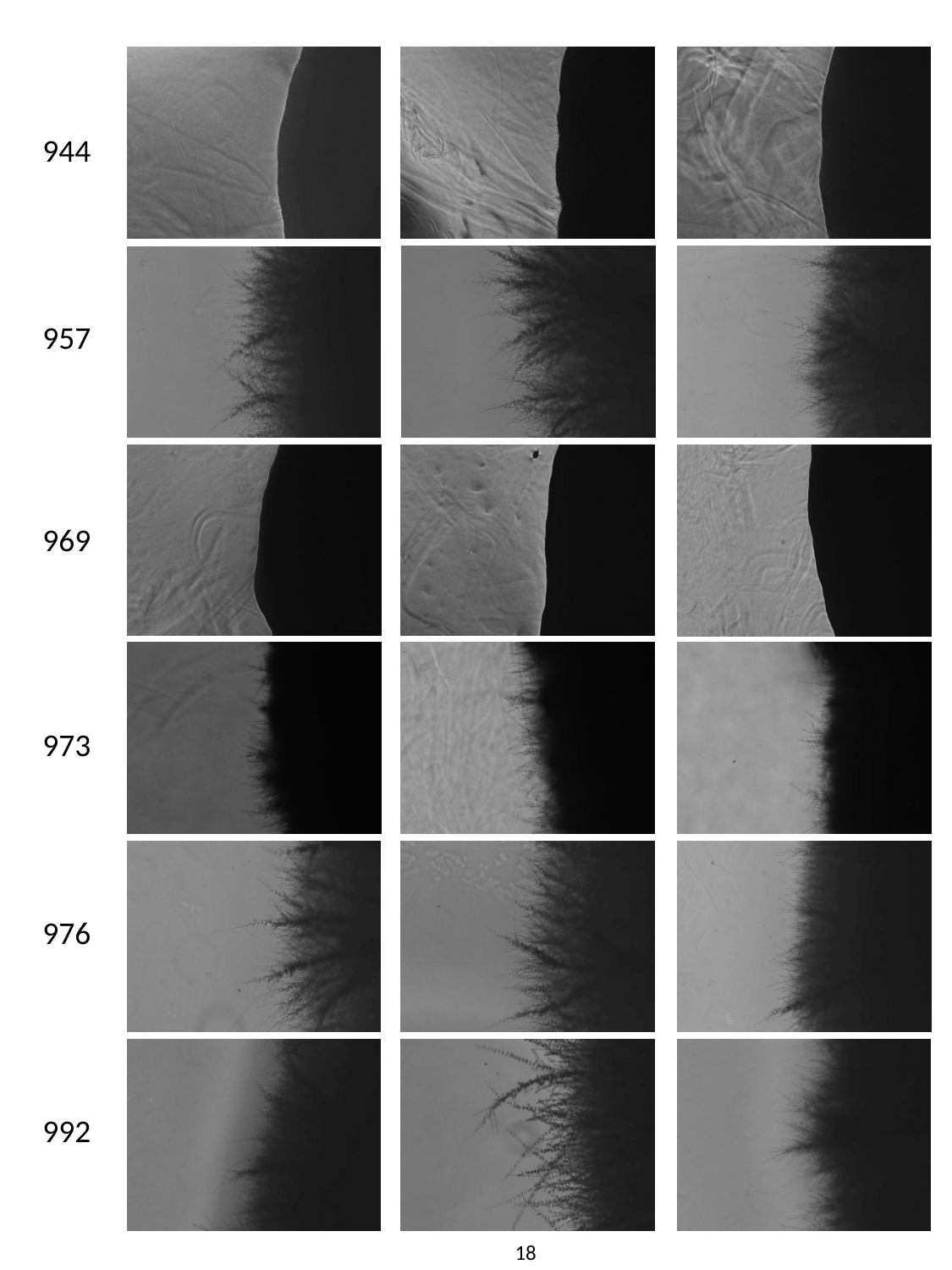

944
957
969
973
976
992
18

## Slide 19
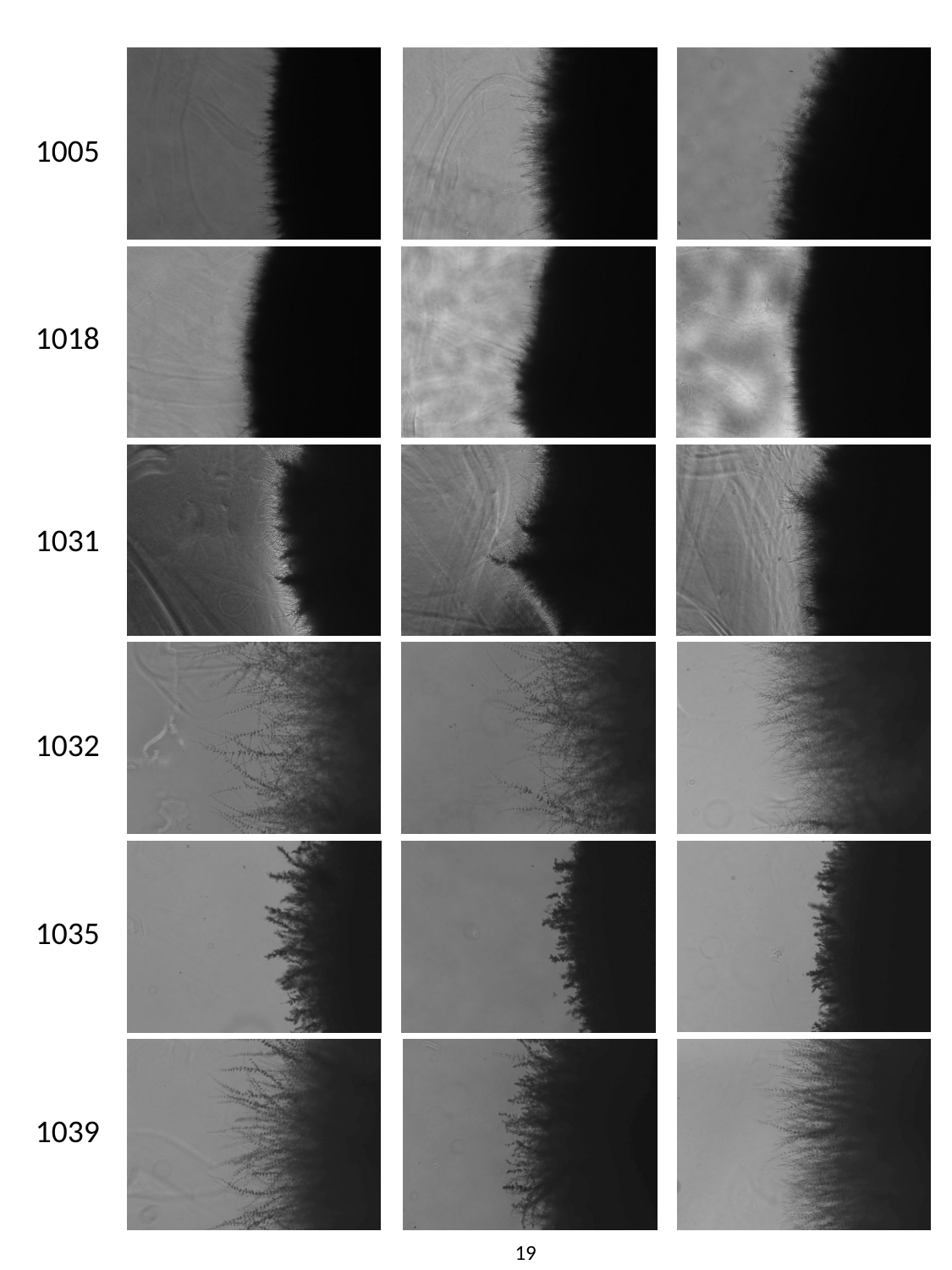

1005
1018
1031
1032
1035
1039
19

## Slide 20
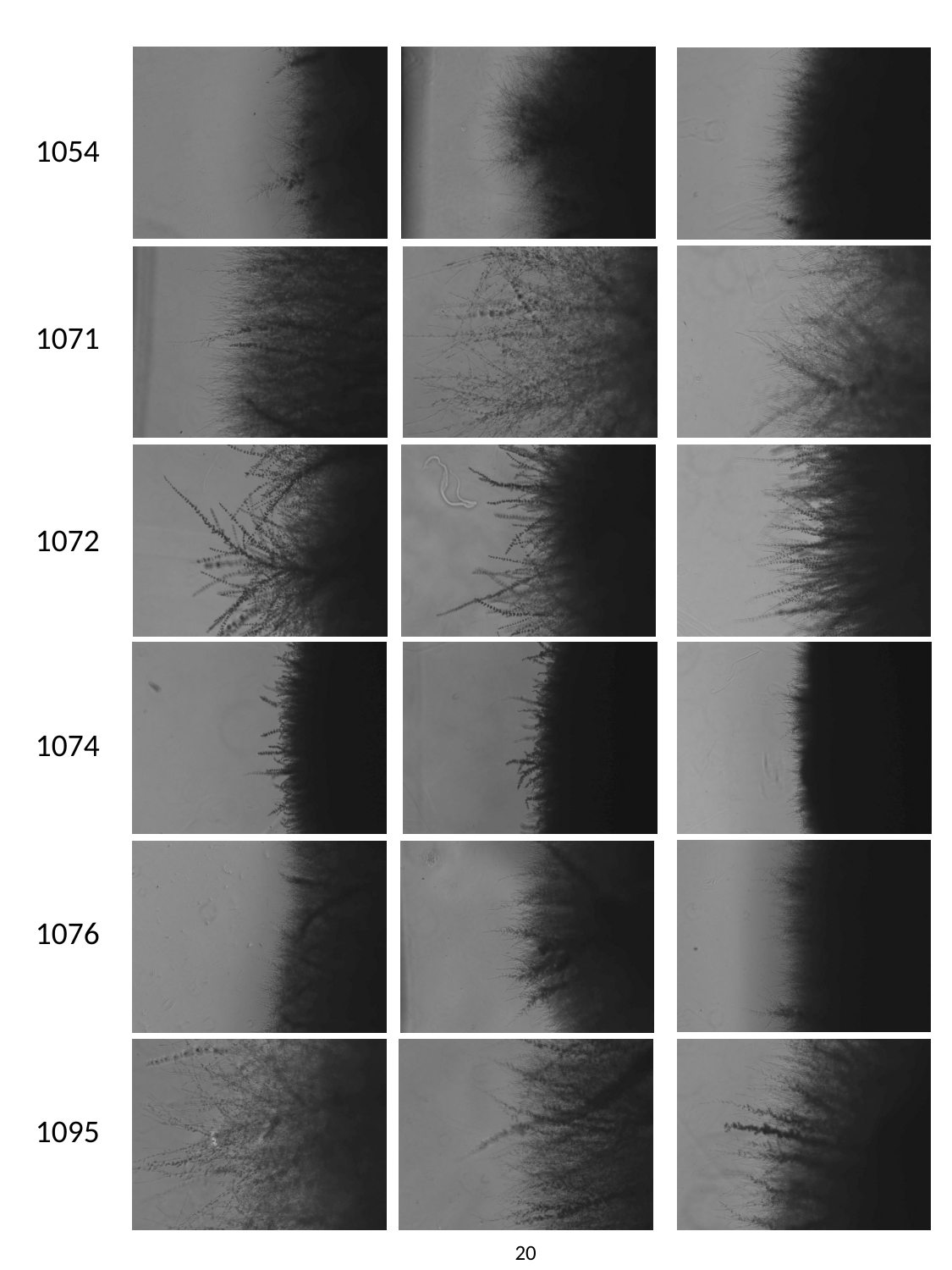

1054
1071
1072
1074
1076
1095
20

## Slide 21
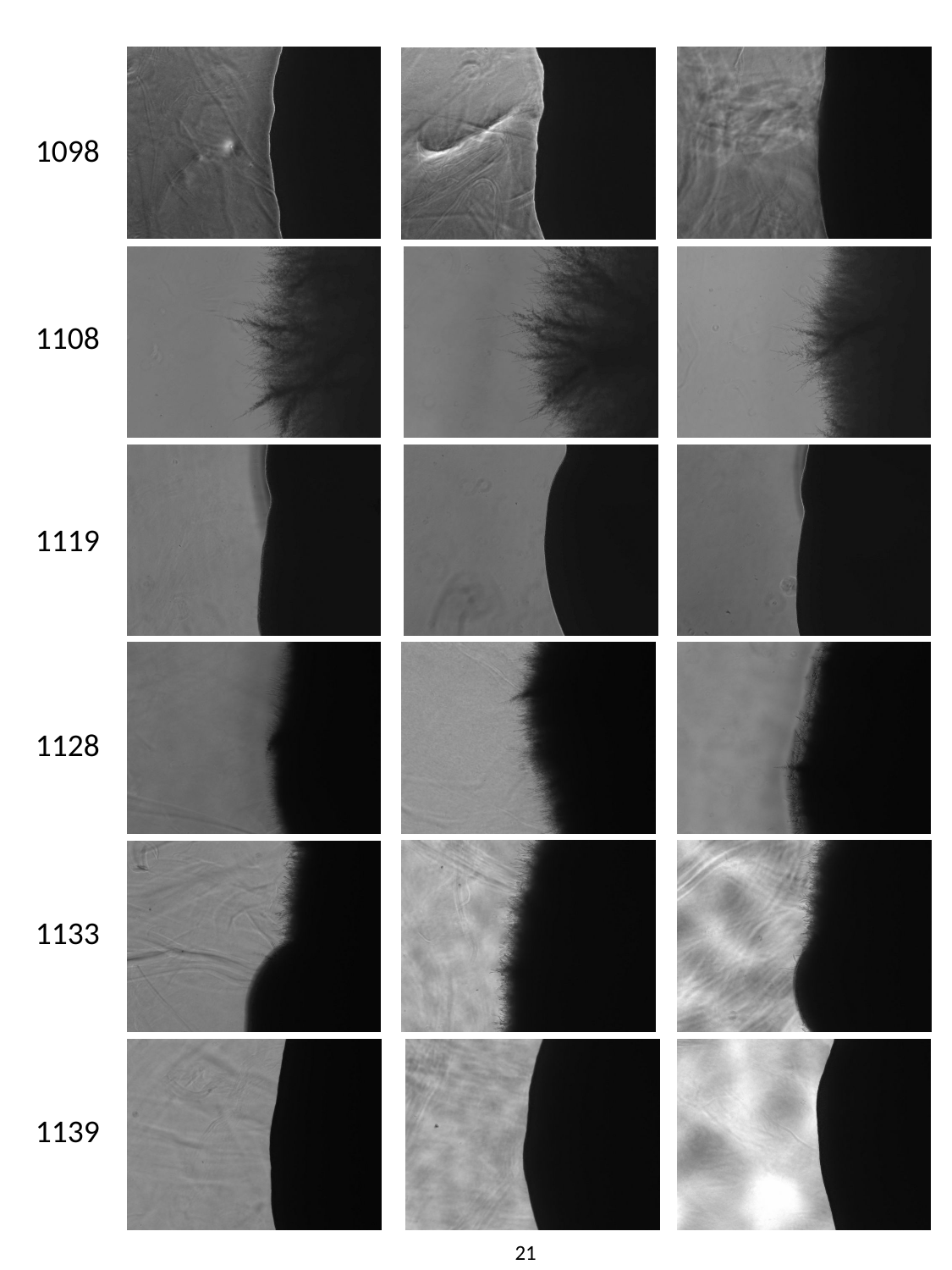

1098
1108
1119
1128
1133
1139
21

## Slide 22
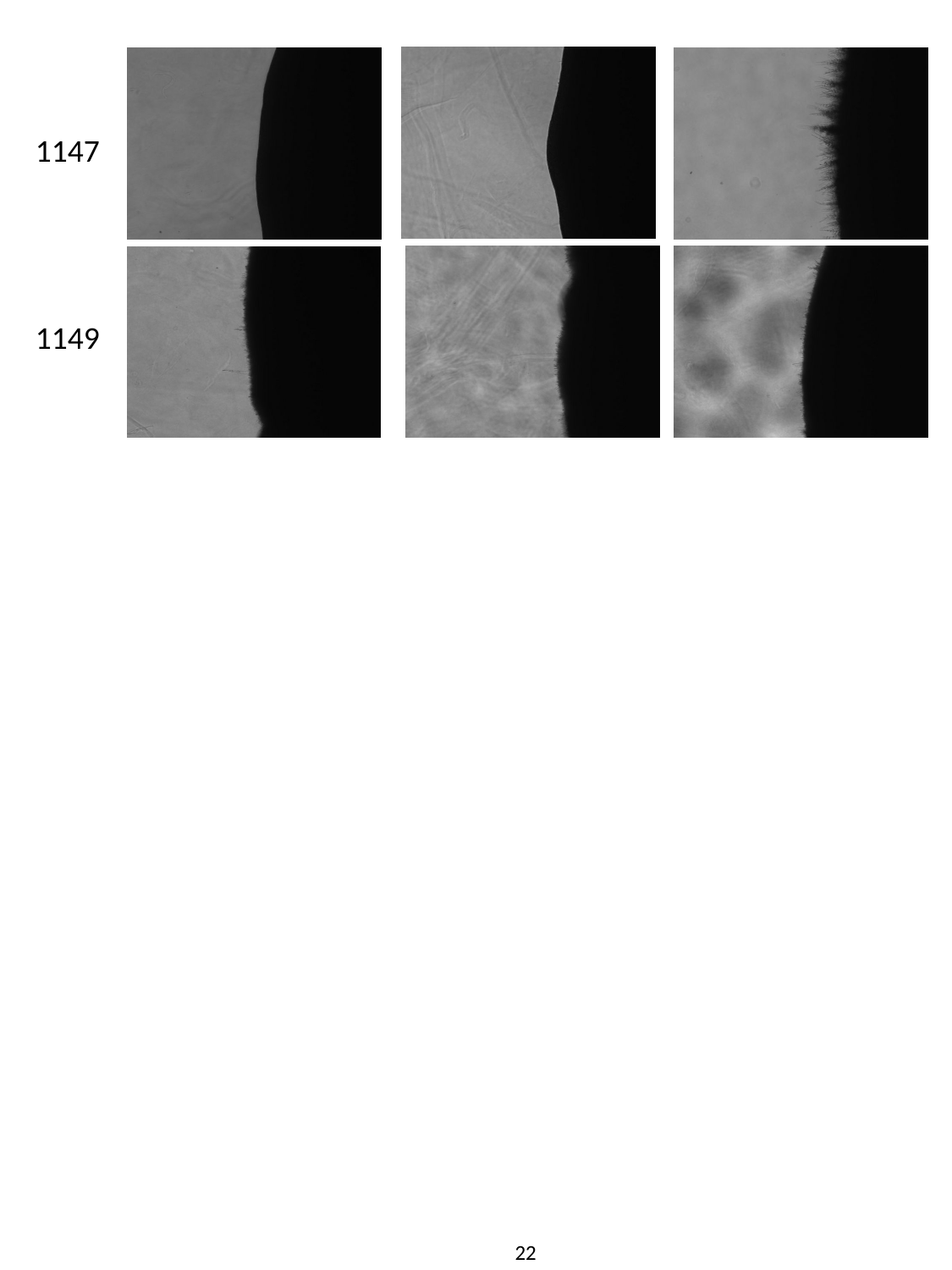

1147
1149
22
