# Supplementary material for: Filamentation Involves Two Overlapping, but Distinct, Programs of Filamentation in the Pathogenic Fungus Candida albicans
Source: G3 (Bethesda). 2017 Sep 25;7(11):3797–808. doi: 10.1534/g3.117.300224 (PMC5677161; doi:10.1534/g3.117.300224)
Supplement: Supplementary file 17 [file 3797FileS1.pdf]

Figure S1. Filamentation assays in solid FBS media. A wild type (SC5314), marker matched (SN250), and strains from the Noble mutant collection (numbered according to their strain number in the collection) were tested for filamentation on solid 10% FBS plates as described in the methods. Images of the colony edge were taken after 4-5 days of induction at 37˚C. Images of each of the triplicate assays, taken on separate plates, are shown. A key for the gene mutated in each strain can be found in Table S6.

Figure S2. Filamentation assays in liquid FBS media. A wild type (SC5314), marker matched (SN250), and strains from the Noble mutant collection (numbered according to their strain number in the collection) were tested for filamentation in liquid 10% FBS + YPD media as described in the methods. Images were taken of cells after 3 hours of induction at 37˚C. 3-5 images are shown for each assay. A key for the gene mutated in each strain can be found in Table S6.

Figure S3. Filamentation assays in solid Lee's media. A wild type (SC5314), marker matched (SN250), and strains from the Noble mutant collection (numbered according to their strain number in the collection) were tested for filamentation on solid Lee's plates as described in the methods. Images of the colony edge were taken after 4-5 days of induction at 37˚C. Images of each of the triplicate assays, taken on separate plates, are shown. A key for the gene mutated in each strain can be found in Table S6.

Figure S4. Filamentation assays in liquid Lee's media. A wild type (SC5314), marker matched (SN250), and strains from the Noble mutant collection (numbered according to their strain number in the collection) were tested for filamentation in liquid Lee's media as described in the methods. Images were taken of cells after 3 hours of induction at 37˚C. 3-5 images are shown for each assay. A key for the gene mutated in each strain can be found in Table S6.

Figure S5. Filamentation assays in solid RPMI media. A wild type (SC5314), marker matched (SN250), and strains from the Noble mutant collection (numbered according to their strain number in the collection) were tested for filamentation on solid RPMI plates as described in the methods. Images of the colony edge were taken after 4-5 days of induction at 37˚C. Images of each of the triplicate assays, taken on separate plates, are shown. A key for the gene mutated in each strain can be found in Table S6.

Figure S6. Filamentation assays in liquid RPMI media. A wild type (SC5314), marker matched (SN250), and strains from the Noble mutant collection (numbered according to their strain number in the collection) were tested for filamentation in liquid RPMI media as described in the methods. Images were taken of cells after 3 hours of induction at 37˚C. 3-5 images are shown for each assay. A key for the gene mutated in each strain can be found in Table S6.

Figure S7. Filamentation assays in solid Spider media. A wild type (SC5314), marker matched (SN250), and strains from the Noble mutant collection (numbered according to their strain number in the collection) were tested for filamentation on solid Spider plates as described in the methods. Images of the colonies were taken after 4-5 days of induction at 37˚C. Images of each of the triplicate assays, taken on separate plates, are shown. A key for the gene mutated in each strain can be found in Table S6.

Figure S8. Filamentation assays in liquid Spider media. A wild type (SC5314), marker matched (SN250), and strains from the Noble mutant collection (numbered according to their strain number in the collection) were tested for filamentation in liquid Spider media as described in the methods. Images were taken of cells after 3 hours of induction at 37˚C. 3-5 images are shown for each assay. A key for the gene mutated in each strain can be found in Table S6.

Figure S9. Filamentation assays in solid YPD media. A wild type (SC5314), marker matched (SN250), and strains from the Noble mutant collection (numbered according to their strain number in the collection) were tested for filamentation on solid YPD plates as described in the methods. Images of the colony edge were taken after 4-5 days of growth at 30˚C. Images of each of the triplicate assays, taken on separate plates, are shown. A key for the gene mutated in each strain can be found in Table S6.

Figure S10. Filamentation assays in liquid YPD media. A wild type (SC5314), marker matched (SN250), and strains from the Noble mutant collection (numbered according to their strain number in the collection) were tested for filamentation in liquid YPD media as described in the methods. Images were taken of cells after overnight cultures grown in YPD at 30˚C with shaking were washed with PBS prior to initiating filamentation assays. 3-5 images are shown for each assay. A key for the gene mutated in each strain can be found in Table S6.

Table S1. Read statistics for RNAseq

Table S2. Averaged blinded phenotype scores for mutant strains.

Table S3. Averaged blinded scores for genes with virulence defects.

Table S4. Log_2 expression data for strains grown in inducing and control conditions.

Table S5. Log_2 expression values for genes differentially regulated in all solid, all liquid, or in all filamentation conditions.

Table S6. Noble collection strain numbers for phenotypic assays.
